# A complex with nitrogen single, double, and triple bonds to the same chromium atom: synthesis, structure, and reactivity†
†Electronic supplementary information (ESI) available. CCDC 1428950–1428953. For ESI and crystallographic data in CIF or other electronic format see DOI: 10.1039/c5sc04608d


**DOI:** 10.1039/c5sc04608d

**Published:** 2016-01-13

**Authors:** Evan P. Beaumier, Brennan S. Billow, Amrendra K. Singh, Shannon M. Biros, Aaron L. Odom

**Affiliations:** a Michigan State University , Department of Chemistry , 578 S. Shaw Ln , East Lansing , MI 48824 , USA . Email: odom@chemistry.msu.edu; b Grand Valley State University , Department of Chemistry , Allendale , MI 49401 , USA

## Abstract

A nitrogen-based analogue of the Schrock and Clark “yl-ene-yne” complex, W(CBu^*t*^)(CHBu^*t*^)(CH_2_Bu^*t*^)(dmpe), has been prepared. The new complex is the nitrido, imido, amido anion [NCr(NPh)(NPr^i^_2_)_2_]^–^, which was structurally characterized with the [K(crypt-2.2.2)]^+^ counterion. The “Cr–N 1-2-3” complex was prepared from NCr(NHPh)(NPr^i^_2_)_2_, which exists as this nitrido–amido tautomer, rather than the bis(imido) Cr(NH)(NPh)(NPr^i^_2_)_2_. By selection of electrophile, the nitrido–imido salt K[NCr(NPh)(NPr^i^_2_)_2_] can undergo reaction at either the imido or the nitrido to form unusual examples of nitrido or bis(imido) complexes.

## Abstract 2

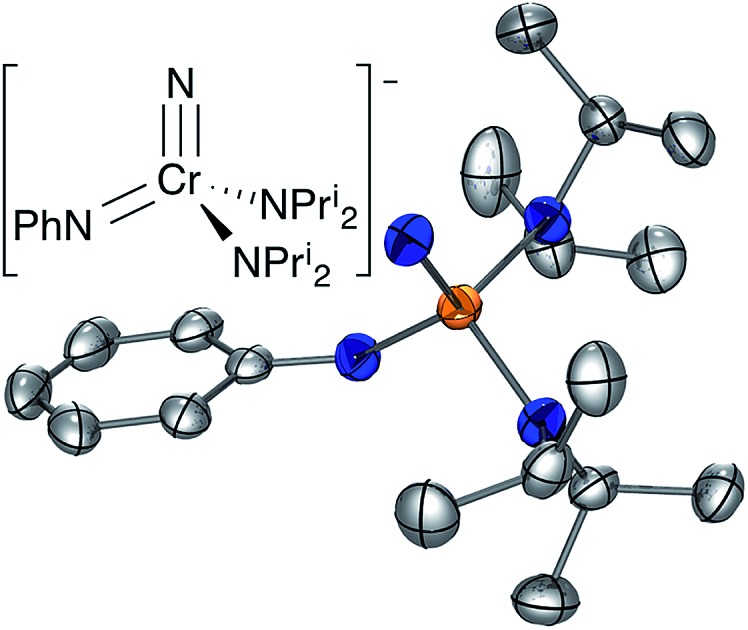
A complex with single, double and triple bonds between nitrogen and the same metal center has been synthesized, [NCr(NPh)(NPr^i^_2_)_2_]^–^. The complex shows differential activity, with some electrophiles attacking the imido and others the nitrido.

## Introduction

**Fig. 1:**
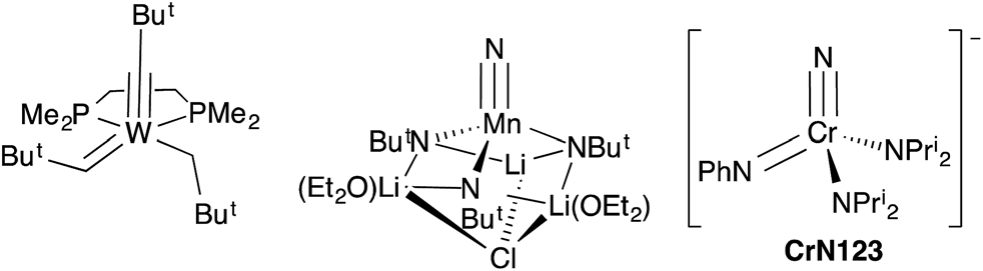
Structures of Schrock and Clark's yl-ene-yne complex,^1^ Wilkinson's nitrido–imido complex (half of the dimer is shown and the chloride bridges to one lithium of a chemically equivalent manganese center),^2^ and the NCr(NPh)(NPr^i^_2_)_2_ (**CrN123**) anion (this work).

In general, synthetic chemists are often fascinated with the unexpected aspects of a chemical transformation with element–element bonds being made or broken. Alternatively, there are compounds that are of interest because they have unusual structures, static pictures with unusual bonding patterns. One complex of the later type often displayed in courses on Organometallic Chemistry is the “yl-ene-yne”, alkyl–alkylidene–alkylidyne, complex W(CBu^*t*^)(CHBu^*t*^)(CH_2_Bu^*t*^)(dmpe) of Clark and Schrock,‡
‡dmpe = 1,2-bis(dimethylphosphino)ethane. which contains metal–carbon single, double and triple bonds to the same tungsten atom (Fig. 1).^1^,^2^

Nitrogen-based complexes containing a variety of multiple bond types in the same complex are less explored. For example, while complexes with multiple imido groups are numerous, there are few examples of terminal nitrido–imido complexes in the Cambridge Structural Database. One example of such a complex is the fascinating manganese-based nitrido complex [Li(OEt_2_)]_2_[MnN(NBu^*t*^)_3_]·LiCl prepared by Danopoulos, Wilkinson, and coworkers with imido ligands that bridge manganese and lithium. The structure is shown schematically in Fig. 1, and is in fact a dimer with chlorides bridging lithium atoms to a chemically identical fragment.^3^

Here, we report the synthesis and structure of the first complex containing nominally nitrogen single, double and triple bonds to the same metal center, [K(crypt-222)][NCr(NPh)(NPr^i^_2_)_2_] (**CrN123** in Fig. 1). The complex is nucleophilic and exhibits differential reactivity, with some electrophiles attacking the imido nitrogen and others the nitride.

## Results and discussion

Our research group has been exploring the synthetic chemistry of chromium nitrido complexes of the general formula NCr(NPr^i^_2_)_2_X, where X = a large host of different substituents, as a means of parameterizing the donor abilities of ligands towards high valent metals. In short, ^1^H NMR spin saturation transfer on the isopropyl groups gives a measure of the donor ability of X. The approximate enthalpy of activation for diisopropylamide rotation, Δ*H*^‡^, has been dubbed the Ligand Donor Parameter (LDP), which grows smaller as X becomes a better donor towards the metal center.^4^

**Fig. 2:**
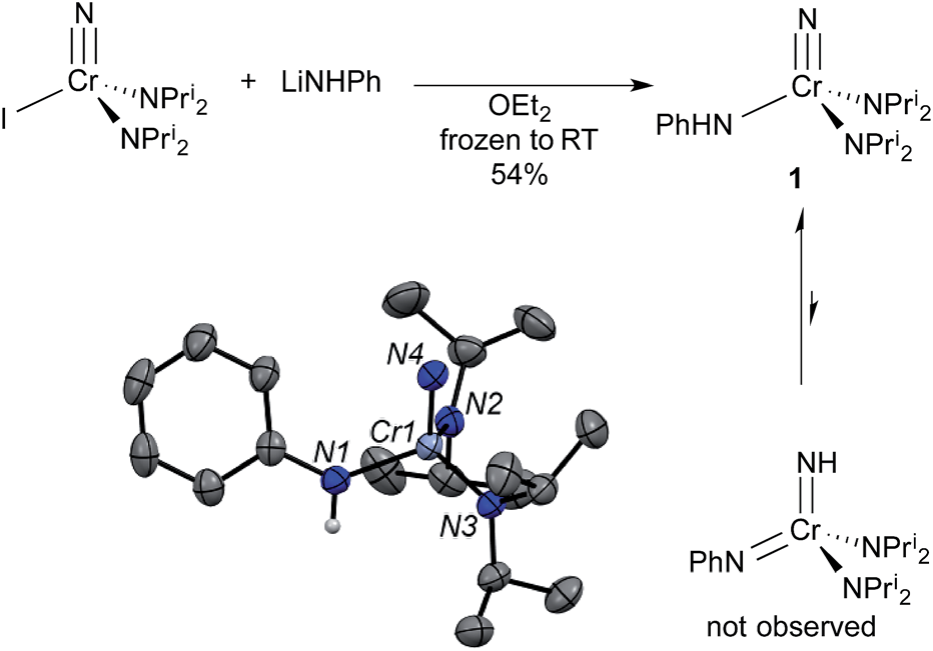
Synthesis and structure from X-ray diffraction of NCr(NPr^i^_2_)_2_(NHPh) (**1**). The bis(imido) tautomer Cr(NH)(NPh)(NPr^i^_2_)_2_ is not observed in solution or the solid state. Ellipsoids are at the 50% probability level and hydrogens on carbon are not shown. Selected bond distances [Å] and angles [°]: Cr1–N1, 1.896(3); Cr1–N2, 1.824(2); Cr1–N3, 1.815(2); Cr1–N4, 1.542(3); N4–Cr1–N1, 107.5(1), N4–Cr1–N2, 104.0(1); N1–Cr1–N2, 110.2(1); N2–Cr–N3, 117.0(1).

One advantage of the NCr(NPr^i^_2_)_2_X system is its synthetic versatility, and dozens of such complexes have been prepared with different X ligands. Treatment of the previously reported iodide complex NCr(NPr^i^_2_)_2_I with LiNHPh gave an unusual NH–anilido nitrido complex NCr(NPr^i^_2_)_2_(NHPh) (**1**). One impetus for the production of such a complex was to examine the possible equilibrium between **1** and the bis(imido) compound Cr(NH)(NPh)(NPr^i^_2_)_2_ (Fig. 2).^3^

In the solid state, complex **1** exists as the nitride anilide, based on the Cr–N(nitrido) and Cr–N(anilido) distances of 1.542(3) and 1.896(3) Å, respectively. If the complex contained Cr=N linkages, the bond distances should be ∼1.7 Å (*vide infra*). The average Cr–NPr^i^_2_ distance is 1.820(3) Å. The NPr^i^_2_–Cr–NPr^i^_2_ angle is significantly larger than the tetrahedral angle at 117.0(1)°.

**Fig. 3:**
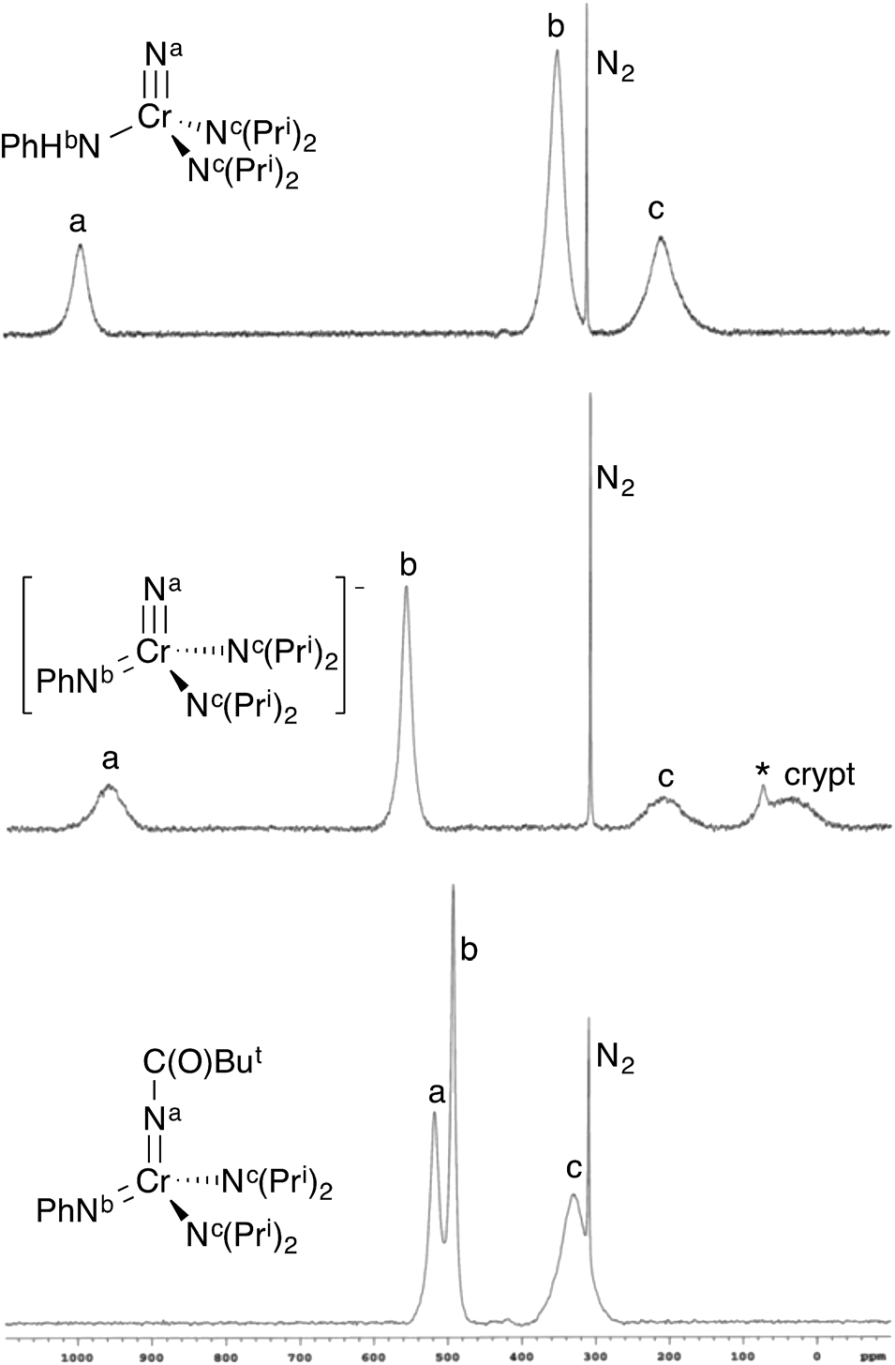
^14^N NMR spectra of some selected complexes. The sharp singlet is dissolved N_2_. Spectra are on the NH_3_ = 0 ppm scale. (top) NCr(NHPh)(NPr^i^_2_)_2_ (**1**) in CDCl_3_. (middle) [K(crypt-2.2.2)][NCr(NPh)(NPr^i^_2_)_2_] (**CrN123**) in *d*_8_-THF. There is a minor impurity (marked with *) at 74 ppm due to HNPr^i^_2_ resulting from trace adventitious water. (bottom) Cr[NC(O)Bu^*t*^](NPh)(NPr^i^_2_)_2_ (**4**) in CDCl_3_. The integrals of the peaks are not necessarily indicative of concentration of the nuclei (see ESI†).

In solution, only one set of septets corresponding to the isopropyl groups of the diisopropylamido ligands is observed in the ^1^H NMR spectrum, consistent with a single species in solution or very fast exchange even at –60 °C. Further, the barrier to diisopropylamido rotation (LDP) in **1** of 9.56 kcal mol^–1^ is essentially identical to NCr(NPr^i^_2_)_2_(NMePh) at 9.57 kcal mol^–1^.§
§The LDP value given here is a more accurate measurement for X = N(Me)Ph than the one given in ref. 3. Errors for the LDP values in this paper are on the order of ±0.2 kcal mol^–1^. If the complex existed as the bis(imido), no observable barrier to amido rotation would be expected. All of this is consistent with **1** being the nitride–anilide NCr(NPr^i^_2_)_2_(NHPh) in solution as well. The solution ^14^N NMR spectrum of **1** is also consistent with a nitride complex (Fig. 3 top). The peak at 991 ppm is characteristic of a chromium(vi) nitrido nitrogen. Conversely, Cr(vi) imido peaks are typically around ∼500 ppm in the nitrogen NMR spectrum (*vide infra*). Also quite apparent are two different amido resonances: one for the anilide at 363 ppm and one for the two diisopropylamides at 182 ppm^5^ (for a discussion of integrals in ^14^N NMR spectra, see the ESI†).

Calculations on complexes **1** and Cr(NPr^i^_2_)_2_(=NPh)(=NH) suggest that the nitrido tris(amido) isomer **1** is enthalpically favored by 18 kcal mol^–1^. A plausible explanation for the nitride structure being lower in energy is that this bis(imido) bis(amido) structure is overloaded with π-donor ligands, *i.e.*, there are more donors than acceptor orbitals leading to electron–electron repulsions that raise the overall energy. In the nitrido tris(amido) tautomer, the nitrido ligand forms a triple bond along with three amides, each with a possible double bond to the metal—5 potential π-bonds total. In the bis(imido) tautomer, each imide can possibly form a triple bond along with double bonds to each amide; 6 potential π-bonds to the ligands. While such π-loading does not preclude stability of the complex and there are many known bis(imido) bis(amido) complexes, it seems in this case the nitrido tris(amido) structure offers increased stability.^6^

**Scheme 1:**
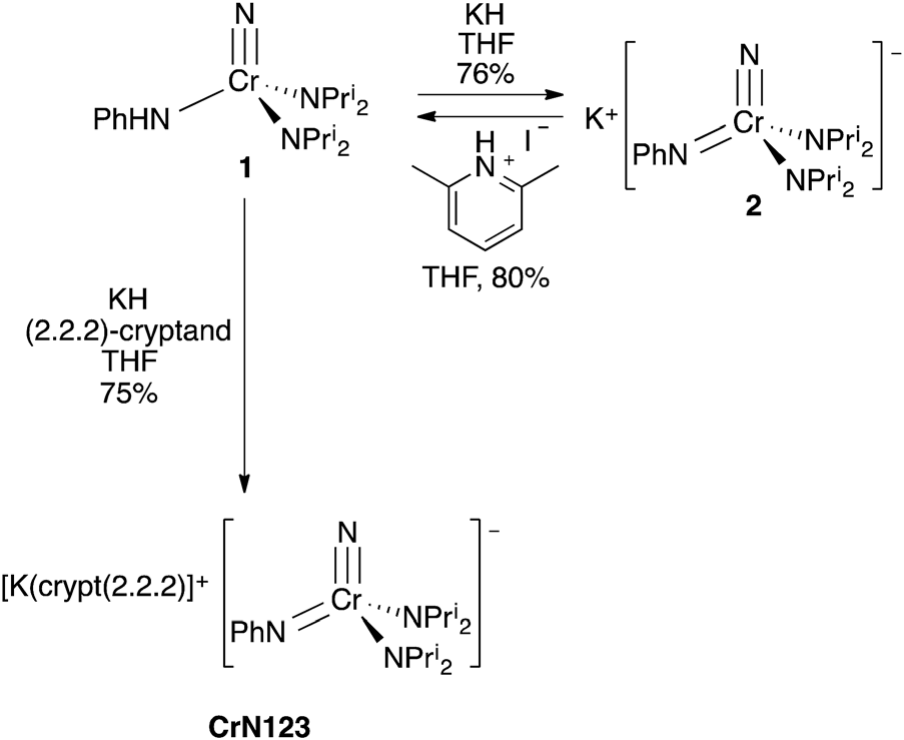
Synthesis of K[NCr(NPh)(NPr^i^_2_)_2_] (**2**) and [K(crypt-222)][NCr(NPh)(NPr^i^_2_)_2_] (**CrN123**) from **1**. Synthesis of **1** from **2** by addition of anhydrous lutidinium iodide.

**Fig. 4:**
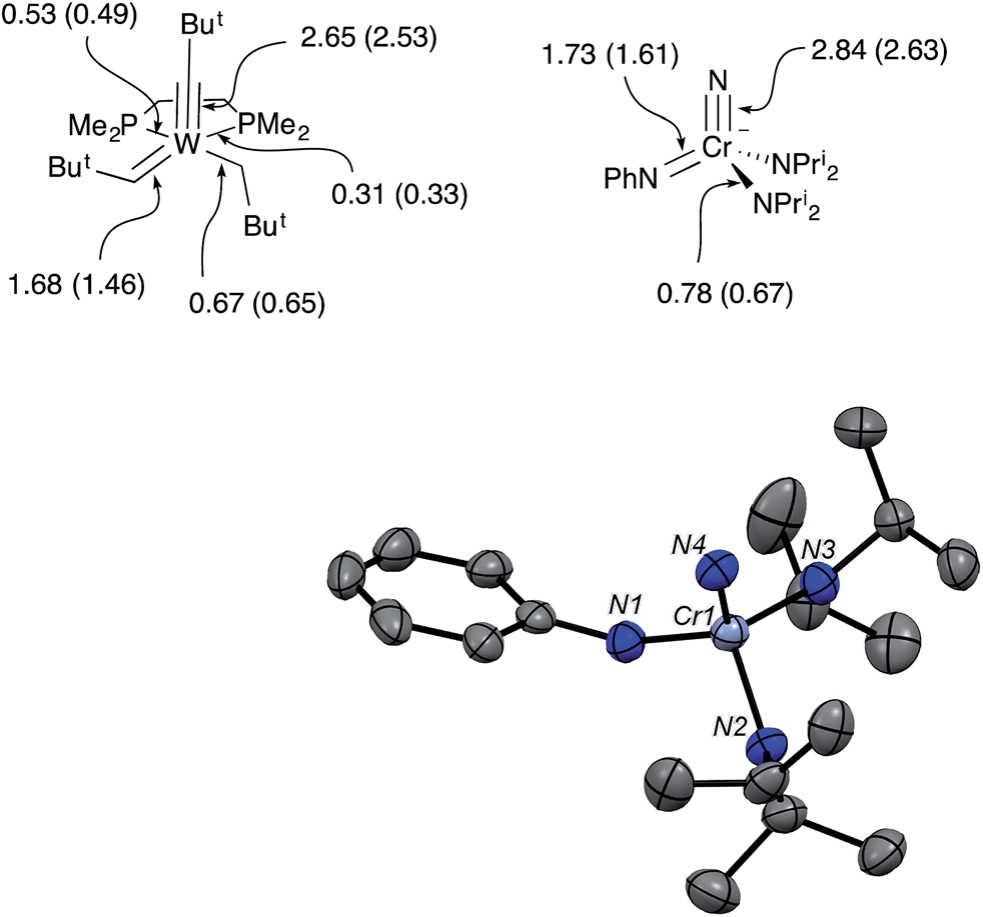
(top) Mayer bond orders and bond orders from local natural resonance theory (in parentheses) for W(CBu^*t*^)(CHBu^*t*^)(CH_2_Bu^*t*^)(dmpe) and the anion of **CrN123**. (bottom) Structure from X-ray diffraction of the anion in [K(crypt-2.2.2)][NCr(NPh)(NPr^i^_2_)_2_] (**CrN123**). Ellipsoids are at the 50% probability level. A disordered pentane molecule in the lattice, cation, and hydrogens in calculated positions are not shown. Selected bond distances [Å] and angles [°]: Cr1–N1, 1.728(3); Cr1–N2, 1.879(3); Cr1–N3, 1.862(2); Cr1–N4, 1.554(3); N4–Cr1–N1, 109.6(1), N4–Cr1–N2, 110.0(1); N1–Cr1–N2, 106.0(1); N2–Cr–N3, 110.3(1).

Treatment of **1** with KH in THF gives K[NCr(NPh)(NPr^i^_2_)_2_] (**2**) in 76% yield as a dark red complex (Scheme 1). The complex is difficult to liberate from THF entirely; however, treatment of **1** with KH and 1 equiv. of (2.2.2)-cryptand in THF sequesters the potassium cation and liberates the anion for structural study. The amber product, [K(crypt-2.2.2)][NCr(NPh)(NPr^i^_2_)_2_] (**CrN123**), was structurally characterized, and the anion is shown in Fig. 4.

In the solid-state structure of **CrN123**, the average Cr–NPr^i^_2_ distance increased significantly over the value in **1** to 1.871(3) Å, consistent with the stronger donor ability of imide relative to phenylamide. The Cr–N(nitrido) distance, however, is the same as **1** within error. The N–Cr–N angles in **CrN123** are all close to the tetrahedral angle and range from 106.0° to 110.8°.

In solution, **CrN123** exhibits fast diisopropylamide rotation consistent with imide being an extremely good donor as would be expected. Using ^14^N NMR spectroscopy one can easily distinguish the three different nitrogens in the complex along with the nitrogen in the cryptand (Fig. 3) at ∼40 ppm. The chemical shift of the nitrido nitrogen is consistent with significant shielding relative to **1** and occurs at 963 ppm. The imido resonance is fairly typical for chromium(vi) at 560 ppm.^5^,^6^

The Mayer bond orders were calculated for Schrock's W(CBu^*t*^)(CHBu^*t*^)(CH_2_Bu^*t*^)(dmpe) and the anion of **CrN123**. The calculated W–C bond orders for the neopentylidyne, neopentylidene and neopentyl groups were 2.65, 1.68 and 0.67, respectively. In the **CrN123** anion, the Cr–N bond orders to the nitrido, imido and amido were calculated as 2.84, 1.73, and 0.78, respectively, and are remarkably similar to the W–C bond orders in the Schrock complex (Fig. 4).^7^,^8^

In addition, Schrock's W(CBu^*t*^)(CHBu^*t*^)(CH_2_Bu^*t*^)(dmpe) and the anion of **CrN123** were examined by local Natural Resonance Theory (NRT) using NBO6, which are also shown parenthetically in Fig. 4. For both the Cr and W complexes, only the metal center and its directly bound atoms were used for the resonance delocalization calculation. Both Mayer bond analysis and NRT are in good agreement for both compounds, suggesting triple, double and single bonds to the metal center. As may be anticipated, the phosphine donors in the tungsten complex display different bond orders, with the phosphine opposite to the stronger *trans*-influencing alkylidene ligand having a lower order bond.

**Fig. 5:**
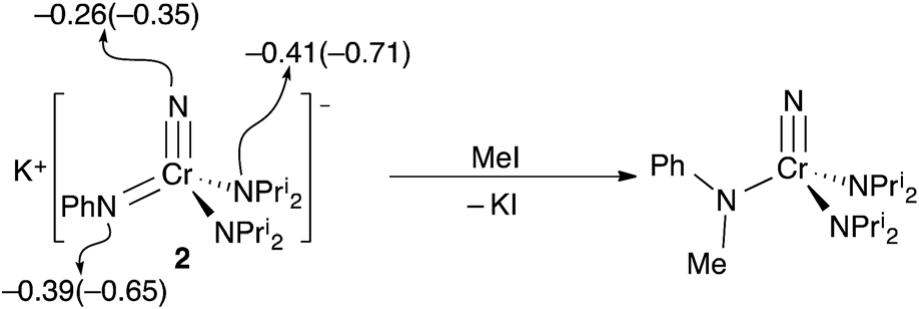
The reaction of **2** with MeI to give the known *N*-methylanilide complex, NCr[N(Me)Ph](NPr^i^_2_)_2_. Calculated Mulliken (NBO) charges for the nitrogens in the [CrN(NPh)(NPr^i^_2_)_2_] anion of **2** are shown. The NPr^i^_2_ charges are the average of the two chemically identical nitrogens.

The reactions of potassium salt **2** with several electrophiles were explored. Since the nitride is a stronger donor than the phenylimide in **2** both Mulliken and NBO charges unsurprisingly suggest that the imide has a larger negative charge, making the imide the charge-preferred site of electrophilic attack, *cf.*, the discussion of **1**–bis(imido) equilibrium above. The preferred site of attack is the imide (Fig. 5) when methyl iodide is used as the electrophile, which gives the known *N*-methylanilido complex, NCr[N(Me)Ph](NPr^i^_2_)_2_.

Based on the structures and charges, we can postulate a reason for nucleophilic attack by imido on methyl iodide (Fig. 5). The preferred site of electrophilic attack in π-delocalized systems is generally the atom with the highest negative charge, *e.g.*, enolate attack by simple electrophiles generally occurs at oxygen.^9^ Amido nitrogen attack is likely prevented by the steric hindrance of the two diisopropyl groups, and the charge on the more sterically accessible imido is preferred. Likewise, addition of acid in the form of anhydrous lutidinium iodide to **2** gave back **1** in 80% yield (Scheme 1).

**Fig. 6:**
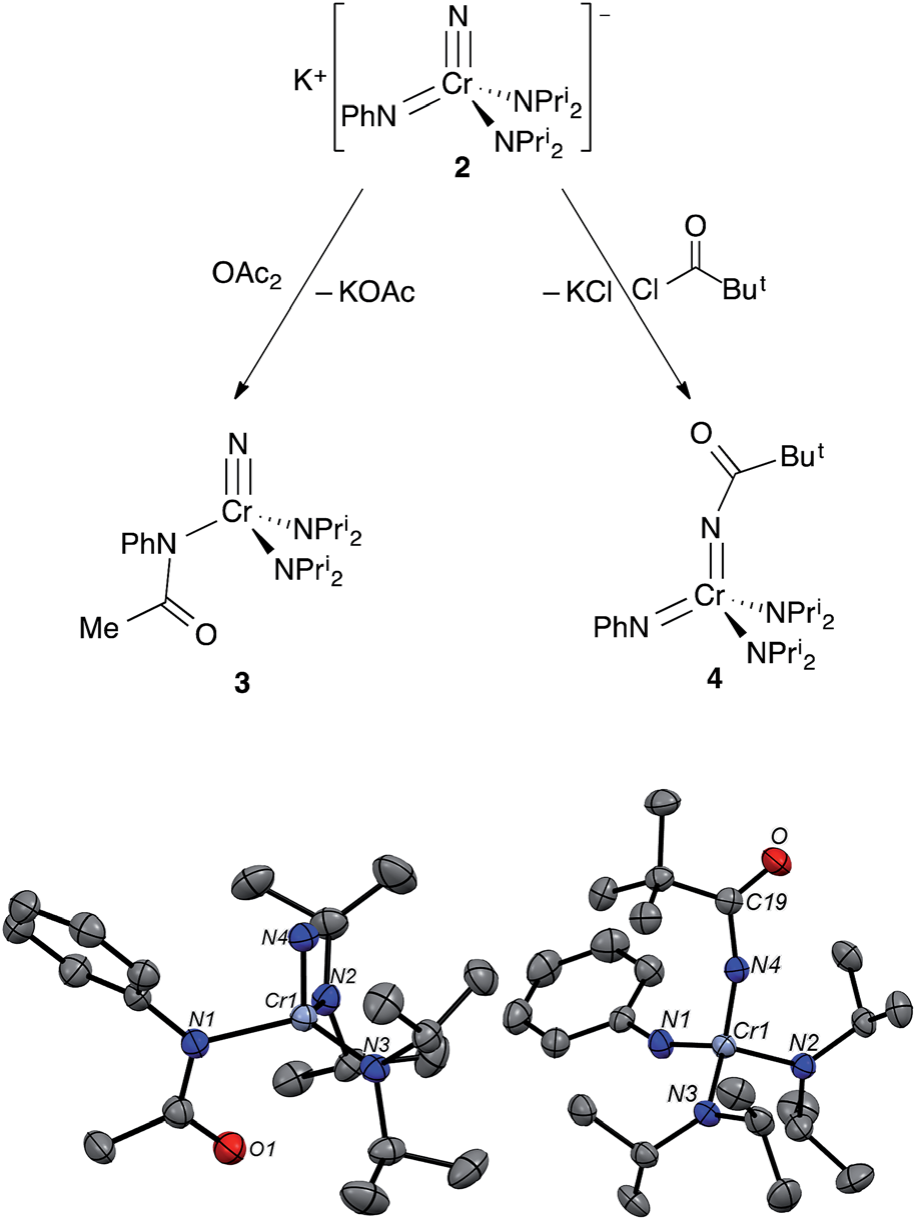
(top) Treatment of **2** with OAc_2_ or ClC(O)Bu^*t*^ results in electrophilic attack at two different sites on the complex to give NCr[N(COMe)Ph](NPr^i^_2_)_2_ (**3**) and Cr[NC(O)Bu^*t*^](NPh)(NPr^i^_2_)_2_ (**4**), respectively. (bottom) Structures from X-ray diffraction of **3** and **4**. Ellipsoids are at the 50% probability level. Only one of the two chemically equivalent molecules in the asymmetric unit is shown, and hydrogens are not shown. Selected bond distances [Å] and angles [°]: (**3**): Cr1–N4, 1.536(2); Cr1–N1, 1.971(2); Cr1–N2, 1.825(2); Cr1–N3, 1.821(2); N1–C19, 1.348(3); N2–Cr1–N3, 117.8(1); N4–Cr1–N3, 103.2(1); N4–Cr1–N2, 103.3(1); N1–Cr1–N3, 112.7(1); N1–Cr1–N2, 112.7(1); N1–Cr1–N4, 98.5(1); (**4**): Cr1–N4, 1.698(2); Cr1–N1, 1.652(2); Cr1–N2, 1.828(2); Cr1–N3, 1.845(2); N4–C19, 1.369(2); O1–C19, 1.226(2); N4–C19, 1.369(2); N2–Cr1–N3, 109.9(1); N4–Cr1–N3, 105.6(1); N4–Cr1–N2, 112.4(1); N1–Cr1–N3, 107.6(1); N1–Cr1–N2, 108.9(1); N1–Cr1–N4, 112.3(1); C19–N4–Cr1, 161.1(2); Cr1–N1–C1, 158.2(2).

Addition of acetic anhydride to **2** (Fig. 6) also leads to attack at the imido nitrogen and provides the nitrido amidinate product NCr[N(COMe)Ph](NPr^i^_2_)_2_ (**3**).¶
¶The product from acyl chloride reaction was the same spectroscopically, but the product was not as clean as with acetic anhydride. That the electrophilic attack occurs at the imide rather than the nitride was definitively assigned by ^14^N NMR; **3** shows a distinct nitride resonance at 1011 ppm along with amidinate and diisopropylamide resonances at 402 and 203 ppm (see the ESI†).

Acyl-containing **3** was also characterized by X-ray diffraction. The structure features a quite long Cr–N(Ph)COMe bond of 1.971(2) Å relative to the diisopropylamide bonds of 1.823(2) Å due to the strongly electron-withdrawing group on the former. That the acyl group strongly interacts with the amido nitrogen can be seen in the short N–C(acyl) bond distance of 1.348(3) Å. There seems to be a weak Cr–O(acyl) interaction as well with a distance of 2.753 Å pseudo-*trans* to the nitride with an N(nitrido)–Cr–O angle of 152°.

An LDP measurement on a crude solution **3** gave a value of 15.09 kcal mol^–1^ for the N(Ph)C(O)Me amidinate fragment. This LDP value, for reference, is similar to chloride (15.05 kcal mol^–1^), but there may be steric influences in the LDP value resulting from the bidentate nature of the ligand, which would raise the LDP over its electronic value.

In contrast, treatment of **2** with pivaloyl chloride leads to reaction at the nitrido nitrogen and formation of the green bis(imide) Cr[NC(O)Bu^*t*^](NPh)(NPr^i^_2_)_2_ (**4**), as shown in Fig. 6. Again, the ^14^N NMR spectrum is definitive for this structure, with two imido resonances at 522 and 493 ppm (Fig. 3). There is no resonance at ∼1000 ppm consistent with a nitrido nitrogen resonance in **4**.

Presumably, the difference in site of attack between ClC(O)Me and ClC(O)Bu^*t*^ is due to the differences in sterics at the electrophile. Because the imido site is more sterically hindered, addition of the sterically encumbered pivaloyl electrophile leads to reaction at the more accessible nitride.

Complex **4** is a rare, structurally characterized example of a transition metal imido complex bearing a carboxyl group on the nitrogen, and the first structurally characterized example of such a complex with chromium^10^,^11^ The Cr–N(imido) distance in the Cr=NC(O)Bu^*t*^ unit is slightly but significantly longer than in the Cr=NPh moiety at 1.688(3) and 1.646(3) Å, respectively. The average Cr–NPr^i^_2_ distance in **4** is similar to **1** at 1.838(3) Å. The N–Cr–N angles are fairly close to tetrahedral and range from 111.6(1)° to 106.3(1)°.

## Concluding remarks

Here, we have described the first example of a complex with nitrogen triple, double and single bonds to the same metal center, **CrN123**. As is often the case, exploration of the syntheses required for the production of the complex led to unusual intermediates as well, and, once formed, the nitrido–imido–amido complex exhibits interesting reactivity commensurate with its unusual structure.

## Supplementary Material

Supplementary information

Crystal structure data
