# Supplementary material for: A complex with nitrogen single, double, and triple bonds to the same chromium atom: synthesis, structure, and reactivity
Source: Chem Sci. 2016 Jan 13;7(4):2532–6. doi: 10.1039/c5sc04608d (PMC5477034; doi:10.1039/c5sc04608d)
Supplement: Supplementary file 1 [file SC-007-C5SC04608D-s001.pdf]

Electronic Supplementary Material (ESI) for Chemical Science.
This journal is © The Royal Society of Chemistry 2016

Supplementary Information:

A Complex with Nitrogen Single, Double, and Triple Bonds to the Same Chromium Atom: Synthesis, Structure, and Reactivity

Evan P. Beaumier[1], Brennan S. Billow[1], Amrendra K. Singh[1], Shannon M. Biros[2] and Aaron L. Odom[1,*]

1. Michigan State University, Department of Chemistry, 578 S. Shaw Ln, East Lansing, MI 48824
2. Department of Chemistry, Grand Valley State University, 1 Campus Dr., Allendale, MI 49401

* Corresponding author e-mail: odom@chemistry.msu.edu

Table of Contents

General Synthetic Considerations 3
Synthesis and Characterization of Compounds 4
$NCr(NPr^{i}_2)_2(NHPh)$ (**1**) 4
$K\{NCr(NPr^{i}_2)_2(NPh)\}$ (**2**) 6
$\{K(crypt\text{-}222)\}\{NCr(NPr^{i}_2)_2(NPh)\}$ (**CrN123**) 6
$NCr(NPr^{i}_2)_2(N(Me)Ph)$ 7
$NCr(NPr^{i}_2)_2[N(COMe)Ph]$ (**3**) 8
$Cr(NPr^{i}_2)_2(NPh)[NC(O)Bu^{t}]$ (**4**) 9
NMR Spectra 10
$NCr(NPr^{i}_2)_2(NHPh)$ (**1**) ^{1}H NMR 10
$NCr(NPr^{i}_2)_2(NHPh)$ ^{13}C NMR 10
$NCr(NPr^{i}_2)_2(NHPh)$ ^{14}N NMR 11
$K\{NCr(NPr^{i}_2)_2(NPh)\}$ (**2**) ^{1}H NMR 12
$K\{NCr(NPr^{i}_2)_2(NPh)\}$ (**2**) ^{14}N NMR 13
$\{K(crypt\text{-}222)\}\{NCr(NPr^{i}_2)_2(NPh)\}$ (**CrN123**) ^{1}H NMR 14
$\{K(crypt\text{-}222)\}\{NCr(NPr^{i}_2)_2(NPh)\}$ (**CrN123**) ^{13}C NMR 14
$\{K(crypt\text{-}222)\}\{NCr(NPr^{i}_2)_2(NPh)\}$ (**CrN123**) ^{14}N NMR 15
$NCr(NPr^{i}_2)_2(N(Me)Ph)$ ^{14}N NMR 16
$NCr(NPr^{i}_2)_2[N(COMe)Ph]$ (**3**) ^{1}H NMR 17

$NCr(NPr^i_2)_2[N(COMe)Ph]$ (**3**) ^{13}C NMR 17
$NCr(NPr^i_2)_2[N(COMe)Ph]$ (**3**) ^{14}N NMR 18
$Cr(NPr^i_2)_2(NPh)[NC(O)Bu^t]$ (**4**) ^{1}H NMR 19
$Cr(NPr^i_2)_2(NPh)[NC(O)Bu^t]$ (**4**) ^{13}C NMR 19
$Cr(NPr^i_2)_2(NPh)[NC(O)Bu^t]$ (**4**) ^{14}N NMR 20
^{14}N NMR of Diisopropylamine in THF 21
^{14}N NMR of 2,2,2-cryptand in $CDCl_3$ 21
Discussion of Peak Integrations in ^{14}N NMR Spectra 22
Method for Determination of Rotational Barriers 22
Table of LDP Measurements 23
Computational Details: 23
References 24

General Synthetic Considerations

All reactions and manipulations were carried out in an MBraun glovebox under a nitrogen atmosphere and using standard Schlenk techniques. Diethyl ether was purchased from Sigma-Aldrich, and pentane was purchased from J.T. Baker. Both were purified through alumina columns to remove water after sparging with dinitrogen to remove dioxygen. Tetrahydrofuran was purchased from Fischer Scientific and distilled over sodium-benzophenone ketyl under dry dinitrogen. $NCr(NPr^i_2)_2(I)$ was prepared as previously reported.[1] Aniline was purchased from Aldrich Chemical Co. and distilled from calcium hydride under reduced pressure then stored under an inert atmosphere. Li-*n*-Bu was purchased from Aldrich Chemical Co. as a 2.5 M solution in hexanes and used as received. Potassium hydride was purchased from Aldrich Chemical Co. as a 30 wt.% dispersion in mineral oil; in a glove box, removal of oil was accomplished by rinsing the dispersion with hexanes. 2,2,2-Cryptand was purchased from Sigma-Aldrich and used as received. Anhydrous lutidinium iodide was prepared using the literature procedure.[2] Methyl iodide was purchased from Sigma-Aldrich and purified by distillation from calcium chloride under dry dinitrogen. Trimethylacetyl chloride (pivaloyl chloride) was purchased from Aldrich Chemical Co. and purified by distillation under reduced pressure. Acetic anhydride was purchased from Columbus Chemical Industries, Inc. and purified by stirring in P_2O_5, followed by distillation from potassium carbonate under reduced pressure.

LiNHPh was prepared by treating a chilled solution of aniline in pentane with 1 equiv. of 2.5 M Bu^nLi in hexanes. The lithium anilide precipitated and was collected in a glass frit then dried in vacuo.

$CDCl_3$, C_6D_6, and THF-d_8 were purchased from Cambridge Isotopes Laboratories, Inc. $CDCl_3$ and C_6D_6 were sparged with dry dinitrogen and dried over 3 Å molecular sieves. THF-d_8 was distilled from sodium-benzophenone ketyl under dry dinitrogen. Spectra were taken on Varian instruments located in the Max T. Rogers Instrumentation Facility at Michigan State University. These include an Agilent DirectDrive2 500 spectrometer equipped with a 5 mm pulsed-field-gradient (PFG) switchable broadband probe and operating at 499.955 MHz (^1H) and 125.77 MHz (^{13}C), as well as a Varian Unity Plus 500 spectrometer with a low gamma broadband probe operating at 36 MHz (^{14}N). NMR chemical shifts are reported in ppm and referenced to the solvent peaks for ^1H NMR ($CDCl_3$, δ 7.26 ppm; C_6D_6, δ 7.16 ppm; THF-d_8, δ 1.72 ppm) and ^{13}C NMR ($CDCl_3$, δ 77.16 ppm; C_6D_6, δ 128.06 ppm; THF-d_8, δ 67.21 ppm). ^{14}N NMR chemical shifts are reported in ppm and referenced to the dinitrogen gas dissolved in solvents (C_6D_6 and THF, δ 309.6 ppm; $CDCl_3$, δ 310.0 ppm), which in turn has been externally referenced against neat CH_3NO_2 as 381.6 ppm. The concentrations for the ^{14}N NMR samples were similar to those used for the ^{13}C NMR data collections, and the collection time was around 12 h. Single crystal X-ray diffraction data was collected in the Center for Crystallographic Research at MSU.

Synthesis and Characterization of Compounds

*$NCr(NPr^i_2)_2(NHPh)$ (**1**)*

Method A: In a 125 mL filter flask equipped with a stir bar was added solid $NCr(NPr^i_2)_2I$ (500 mg, 1.27 mmol, 1 equiv) and 40 mL of ether. The solution was frozen in a liquid nitrogen temperature cold well and allowed to thaw on a stir plate until the solution became a slurry. LiNHPh (377.8 mg, 3.81 mmol, 3 equiv) was dissolved in 10 mL of ether and added dropwise to the cold, stirring solution over a few min. The solution warmed to

room temperature and stirred for 4 h, in which time the solution had gone from orange-red to dark purple. After stirring, the volatiles were removed in vacuo to give a dark purple solid. The resulting solid was extracted with pentane and filtered through Celite in a glass frit. Volatiles were removed in vacuo to afford the complex as a black solid, which was recrystallized from a minimal amount of pentane at –35 °C overnight, affording the product as black plates (244 mg, 54%). *Method B*: In a 20 mL scintillation vial equipped with a stir bar was added solid $K\{NCr(NPr^i_2)_2(NPh)\}$ (**2**, 50 mg, 0.13 mmol, 1 equiv) and 4 mL of THF. The solution was frozen in a liquid nitrogen temperature cold well and allowed to thaw on a stir plate until the solution began to stir. Anhydrous lutidinium iodide (28 mg, 0.12 mmol, 0.95 equiv) was dissolved in 2 mL THF and added dropwise to the cold, stirring solution. The solution quickly went from dark red to dark purple in color, and was allowed to warm to room temperature and stir vigorously for 1 h after addition. After stirring, the volatiles were removed in vacuo to give a dark solid. The resulting solid was extracted with pentane and filtered through Celite. The volatiles were removed in vacuo, yielding the complex as a black powder (34 mg, 80%). The black powder was redissolved in a minimal amount of pentane and stored at –35 °C overnight, affording the recrystallized product as black plates. ^{1}H NMR (500 MHz, $CDCl_3$, 25 °C): δ = 7.12 (t, J_{HH} = 7.7, 2H, Ar-C-*H*), 7.03 (d, J_{HH} = 8.0, 2H, Ar-C-*H*), 6.67 (t, J_{HH} = 8.5,1H, Ar-C-*H*), 6.60 (br s, 1H, Ar-N-*H*), 4.20 (app v br s, 4H, $CH(CH_3)_2$), 1.44 (app v br s, 12 H, $CH(CH_3)_2$), 1.34 (br d, J_{HH} = 5.6, 12H, $CH(CH_3)_2$). $^{13}C\{^1H\}$ NMR (126 MHz, $CDCl_3$, 25 °C) δ = 156.32, 128.73, 118.04, 116.37, 56.12, 56.22 (br s), 45.32 , 29.84, 25.84 (v br s), 23.56. ^{14}N NMR (36 MHz, $CDCl_3$, 25 °C): δ = 991.09 ($\Delta\nu_{1/2}$ = 770.82 Hz), 363.04 ($\Delta\nu_{1/2}$ = 786.47 Hz), 182.10 ($\Delta\nu_{1/2}$ = 894.13 Hz). M.p.: 104-106 °C (dec). Anal. Found (Calcd) for $C_{18}H_{34}N_4Cr$: C, 59.88 (60.31); H, 9.83 (9.56); N, 15.69 (15.63).

*K{NCr(NPr$^i_2$)$_2$(NPh)} (**2**)*

In a 20 mL scintillation vial equipped with a stir bar was added solid NCr(NPr$^i_2$)$_2$(NHPh) (**1**, 82 mg, 0.23 mmol, 1 equiv) and 3 mL of THF. The solution was frozen in a liquid nitrogen temperature cold well and allowed to thaw on a stir plate until the solution began to stir. Potassium hydride (10.1 mg, 0.25 mmol, 1.1 equiv) in 2 mL of THF was added dropwise to the cold, stirring solution. The solution was allowed to warm to room temperature and stirred vigorously for 2 h, over which time the solution went from dark purple to dark red. The volatiles were removed in vacuo to give a dark red oily residue. The residue was extracted with ether and filtered through Celite. Volatiles were removed, and the resulting crude product was redissolved in a minimal amount of ether, layered with pentane, and stored at –35 °C overnight, affording the recrystallized product as a black crystalline solid (69 mg, 76%). ^{1}H NMR (500 MHz, THF-d$_8$, 25 °C): δ = 7.01 (t, J_{HH} = 7.8, 2H, Ar-C-*H*), 6.52 (d, J_{HH} = 7.2, 2H, Ar-C-*H*), 6.35 (t, J_{HH} = 6.8, 1H, Ar-C-*H*), 3.96 (sept, J_{HH} = 6.3, 4H, C*H*(CH$_3$)$_2$), 1.24 (d, J_{HH} = 6.9, 12 H, CH(C*H*$_3$)$_2$), 1.16 (d, J_{HH} = 5.9, 12H, CH(C*H*$_3$)$_2$). ^{13}C{^{1}H} NMR (126 MHz, THF-d$_8$, 25 °C) δ = 170.43, 128.61, 116.08, 113.90, 54.37, 26.01, 25.04. ^{14}N NMR (36 MHz, THF, 25 °C): δ = 933.95 ($\Delta\nu_{1/2}$ = 1111.27 Hz), 545.69 ($\Delta\nu_{1/2}$ = 352.74 Hz), 223.61 ($\Delta\nu_{1/2}$ = 1150.87 Hz). M.p.: 108-110 °C. Anal. Found (Calcd) for $C_{18}H_{33}N_4CrK$: C, 54.21 (54.51); H, 8.36 (8.39); N, 13.41 (14.13).

*{K(crypt-222)}{NCr(NPr$^i_2$)$_2$(NPh)} (**CrN123**)*

In a 20 mL scintillation vial equipped with a stir bar was added solid NCr(NPr$^i_2$)$_2$(NHPh) (**1**, 50 mg, 0.14 mmol, 1 equiv), 2,2,2-cryptand (58 mg, 0.15 mmol, 1.1 eq), and 3 mL of THF. The solution was frozen in a liquid nitrogen temperature cold well

and allowed to thaw on a stir plate until the solution began to stir. Potassium hydride (6.2 mg, 0.15 mmol, 1.1 equiv) in 2 mL of THF was added dropwise to the cold, stirring solution. The solution was allowed to warm to room temperature and stir vigorously for 2 h, over which time the solution went from dark purple to amber. The volatiles were removed in vacuo to give a dark orange solid. The resulting solid was extracted with THF and filtered. The volatiles were removed in vacuo. The solution was redissolved in a minimal amount of THF, layered with pentane, and stored at –35 °C overnight, affording the recrystallized product as dark orange crystals (80 mg, 75%). Adequate elemental analysis was not obtained of the material after several attempts due to its apparent thermal instability and high sensitivity to moisture. ^{1}H NMR (500 MHz, THF-d_8, 25 °C): δ = 6.83 (t, J_{HH} = 7.9, 2H, Ar-C-*H*), 6.64 (d, J_{HH} = 7.5, 2H, Ar-C-*H*), 6.18 (t, J_{HH} = 7.3, 1H, Ar-C-*H*), 3.90 (sept, J_{HH} = 6.4, 4H, C*H*(CH_3)$_2$), 3.52 (s, 12H, OCH_2CH_2O), 3.48 (t, J_{HH} = 4.3, 12H, CH_2O), 2.48 (t, J_{HH} = 4.3, 12H, CH_2N), 1.23 (d, J_{HH} = 6.3, 12H, CH(CH_3)$_2$) 1.13 (d, J_{HH} = 6.3, 12H, CH(CH_3)$_2$). ^{13}C{^{1}H} NMR (126 MHz, THF-d_8, 25 °C) δ = 170.17, 127.19, 114.91, 114.80, 71.10, 68.27, 54.57, 54.16, 26.16, 25.18. ^{14}N NMR (36 MHz, THF, 25 °C): δ = 963.25 ($\Delta\nu_{1/2}$ = 1271.06 Hz), 559.61 ($\Delta\nu_{1/2}$ = 558.98 Hz), 214.33 ($\Delta\nu_{1/2}$ = 1606.00 Hz), 34.86 ($\Delta\nu_{1/2}$ = 2702.92 Hz). M.p.: dec. 114-116 °C followed by melting at 142-144 °C.

NCr(NPr$^{i}_{2}$)$_2$(N(Me)Ph)

In a 20 mL scintillation vial equipped with a stir bar was added solid K{NCr(NPr$^{i}_{2}$)$_2$(NPh)} (**2**, 35 mg, 0.09 mmol, 1 equiv) and 3 mL of ether. The solution was frozen in a liquid nitrogen temperature cold well and allowed to thaw on a stir plate until the solution began to stir. To the cold, stirring solution was introduced methyl iodide (11 μL, 0.18 mmol, 2 equiv). The solution was stirred for 2 h, over which time the solution went

from dark red to purple. Volatiles were removed in vacuo to give a dark purple solid. The resulting solid was extracted with pentane and filtered through Celite. The solution was concentrated and stored at –35 °C overnight, affording the recrystallized product as black needles (26 mg, 79%). The complex matched the previously characterized *N*-methylanilide by ^{1}H NMR spectroscopy and unit cell from X-ray diffraction. ^{14}N NMR (36 MHz, $CDCl_3$, 25 °C): δ = 993.04 ($\Delta\nu_{1/2}$ = 786.22 Hz), 349.36 ($\Delta\nu_{1/2}$ = 830.71 Hz), 207.25 ($\Delta\nu_{1/2}$ = 1356.39 Hz).

*$NCr(NPr^i_2)_2[N(COMe)Ph]$ (**3**)*

In a 20 mL scintillation vial equipped with a stir bar was added solid $K\{NCr(NPr^i_2)_2(NPh)\}$ (**2**, 25 mg, 0.06 mmol, 1 equiv) and 3 mL of ether. The solution was frozen in a liquid nitrogen temperature cold well and allowed to thaw on a stir plate until the solution began to stir. Acetic anhydride (4.5 µL, 0.05 mmol, 0.75 equiv) was introduced to the cold, stirring solution. The solution was allowed to warm to room temperature and stir for 10 min, over which time the solution went from dark red to mauve. Volatiles were removed in vacuo to give a light purple oil. The resulting oil was extracted with pentane and filtered through Celite. Volatiles were removed in vacuo. The residual oil was determined spectroscopically to be consistent with impure **3**. X-ray quality crystals were obtained by slow cooling of a concentrated solution in pentane; however, the sought after product always had thick oily coating on the solids that could not be avoided, which is likely a result of decomposition. Due to this oily residue, satisfactory elemental analysis of **3** was not obtained. ^{1}H NMR (500 MHz, $CDCl_3$, 25 °C): δ = 7.28 (app t, J_{HH} = 8.2, 2H, Ar-C-*H*), 7.09 (dd, J_{HH} = 11.7, 4.2, 1H, Ar-C-H), 7.06 (dt, J_{HH} = 8.1, 2H, Ar-C-*H*), 5.85 (sept, J_{HH} = 6.5, 2H, $CH(CH_3)_2$), 3.84 (sept, J_{HH} = 6.3, 2H, $CH(CH_3)_2$), 1.81 (d, J_{HH} = 6.2, 6H, $CH(CH_3)_2$), 1.73 (s, 3H,

$CH_3C(O)$), 1.53 (d, J_{HH} = 6.2, 6H, $CH(CH_3)_2$), 1.24 (d, J_{HH} = 6.2, 6H, $CH(CH_3)_2$), 1.10 (d, J_{HH} = 6.2, 6H, $CH(CH_3)_2$). $^{13}C\{^1H\}$ NMR (126 MHz, $CDCl_3$, 25 °C) δ = 176.64, 154.29, 128.72, 125.88, 124.70, 57.46, 55.98, 31.03, 29.80, 23.34, 22.07, 21.62. ^{14}N NMR (36 MHz, $CDCl_3$, 25 °C): δ = 1011.11 ($\Delta\nu_{1/2}$ = 656.41 Hz), 401.62 ($\Delta\nu_{1/2}$ = 807.141 Hz), 202.85 ($\Delta\nu_{1/2}$ = 1264.46 Hz). M.p.: 64-67 °C

*$Cr(NPr^i_2)_2(NPh)[NC(O)Bu^t]$ (**4**)*

In a 20 mL scintillation vial equipped with a stir bar was added solid $K\{NCr(NPr^i_2)_2(NPh)\}$ (**2**, 50 mg, 0.13 mmol, 1 equiv) and 3 mL of ether. The solution was frozen in a liquid nitrogen temperature cold well and allowed to thaw on a stir plate until the solution began to stir. To the cold, stirring solution was introduced trimethylacetyl chloride (16 μL, 0.13 mmol, 1 equiv). The solution stirred for 30 min, over which time the solution went from dark red to emerald green. Volatiles were removed in vacuo to give a dark green oily residue. The residue was extracted with pentane and filtered through Celite. The solution was concentrated to a minimal amount and stored at –35 °C overnight, affording the product as a dark green crystalline solid (51 mg, 91%). ^1H NMR (500 MHz, C_6D_6, 25 °C): δ = 7.39 (d, J_{HH} = 7.9, 2H, Ar-C-*H*), 6.98 (t, J_{HH} = 7.8, 2H, Ar-C-*H*), 6.73 (t, J_{HH} = 7.8, 1H, Ar-C-*H*), 4.13 (sept, J_{HH} = 6.5, 4H, $CH(CH_3)_2$), 1.31 (s, 9H, $NC(O)(CH_3)_3$), 1.28 (app t, J_{HH} = 7.0, 24H, $CH(CH_3)_2$). $^{13}C\{^1H\}$ NMR (126 MHz, C_6D_6, 25 °C) δ = 190.05, 163.79, 128.87, 126.47, 124.31, 59.13, 40.71, 28.72 , 25.48, 24.35. ^{14}N NMR (36 MHz, $CDCl_3$, 25 °C): δ = 518.33 ($\Delta\nu_{1/2}$ = 591.43 Hz), 493.05 ($\Delta\nu_{1/2}$ = 264.16 Hz), 328.84 ($\Delta\nu_{1/2}$ = 1608.68 Hz). M.p.: 77-79 °C (dec). Anal. Found (Calcd) for $C_{23}H_{42}N_4OCr$: C, 61.95 (62.41); H, 9.20 (9.56); N, 12.40 (12.66).

NMR Spectra

*NCr(NPr$^{i}_{2}$)$_2$(NHPh) (**1**) ^{1}H NMR*

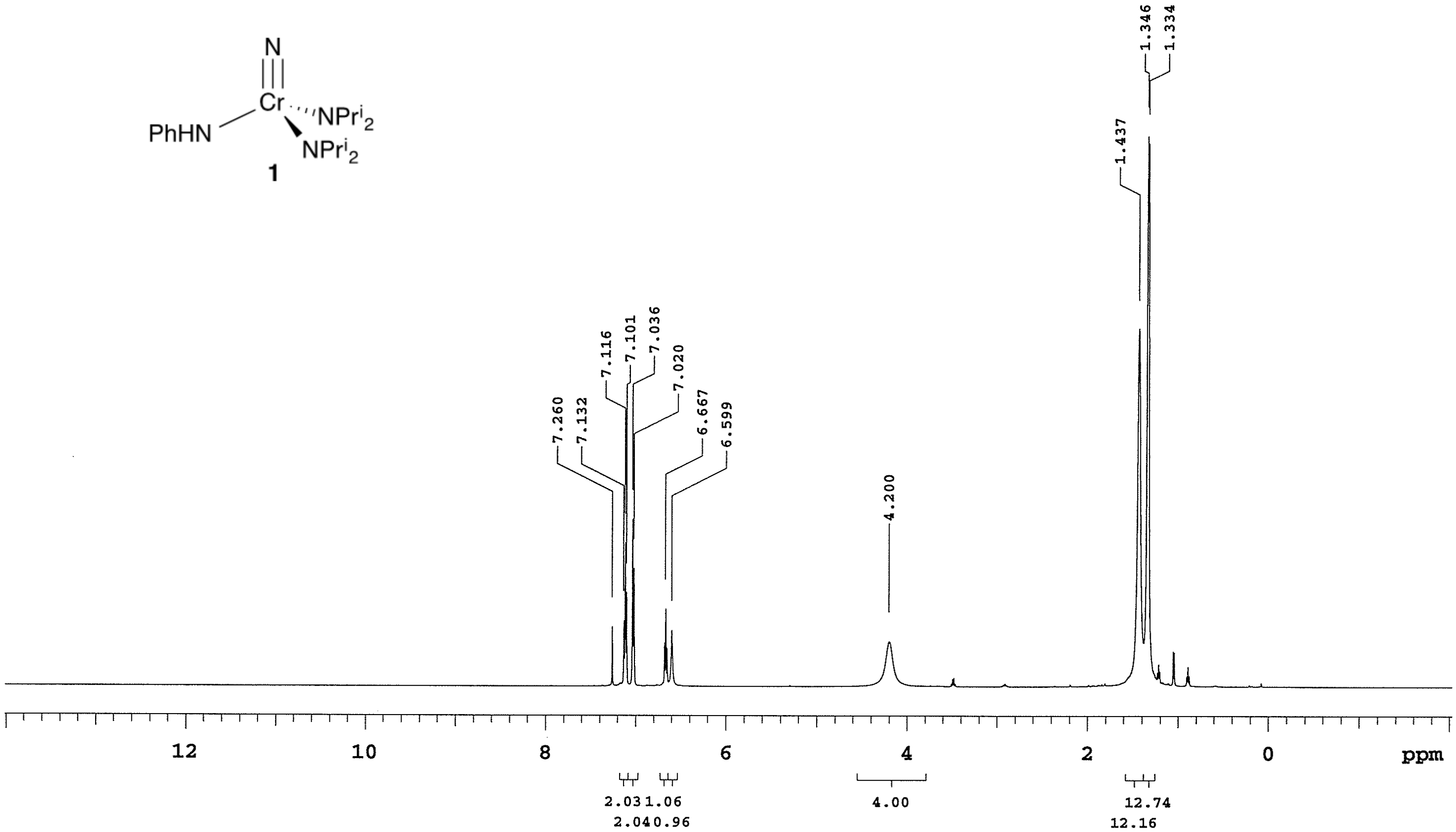

NCr(NPr$^{i}_{2}$)$_2$(NHPh) ^{13}C NMR

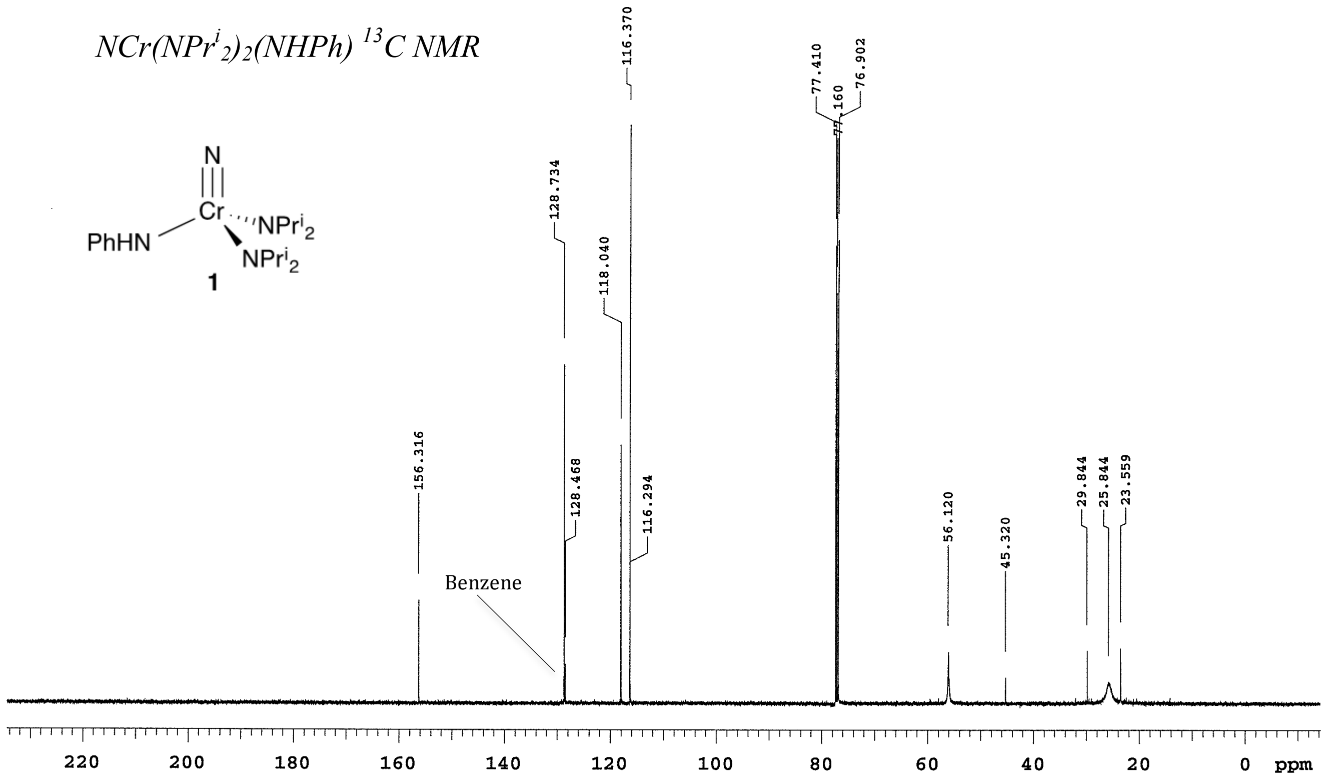

NCr(NPr$^{i}_{2}$)$_2$(NHPh) ^{14}N NMR

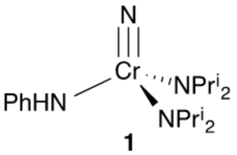

1

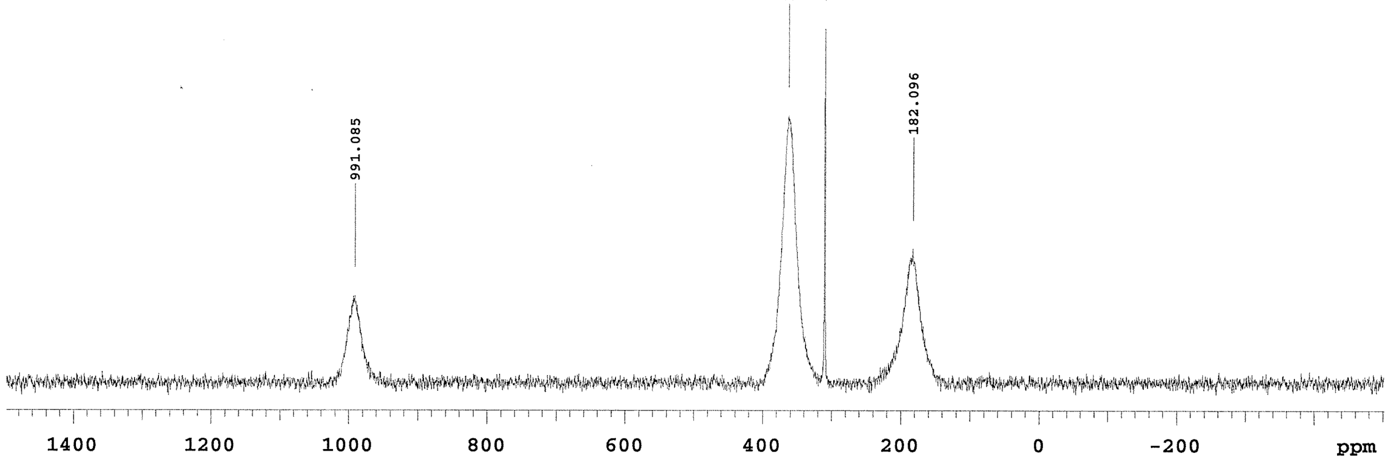

*K{NCr(NPr$^{i}_{2}$)$_2$(NPh)} (**2**) ^{1}H NMR*

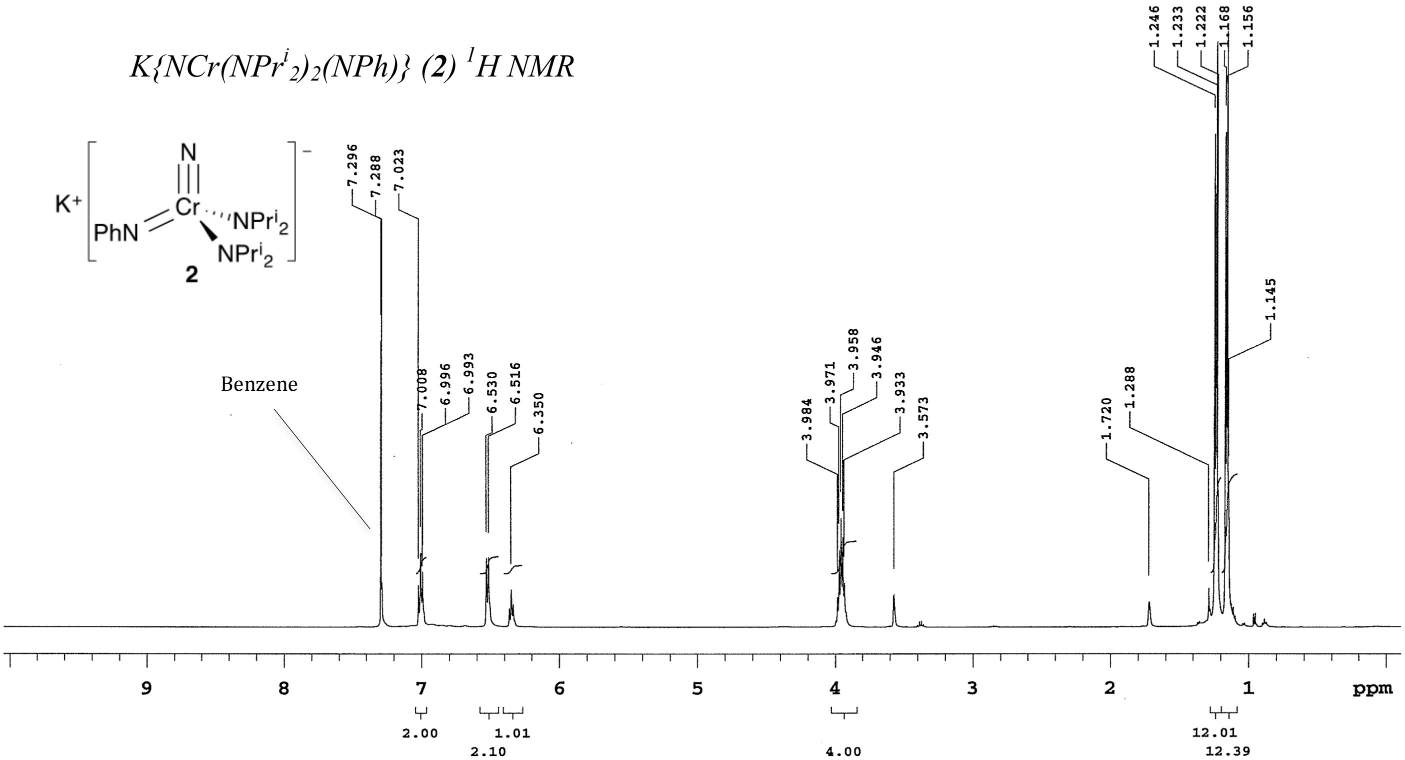

K{NCr(NPr$^{i}_{2}$)$_2$(NPh)} (***2***) ^{13}C NMR

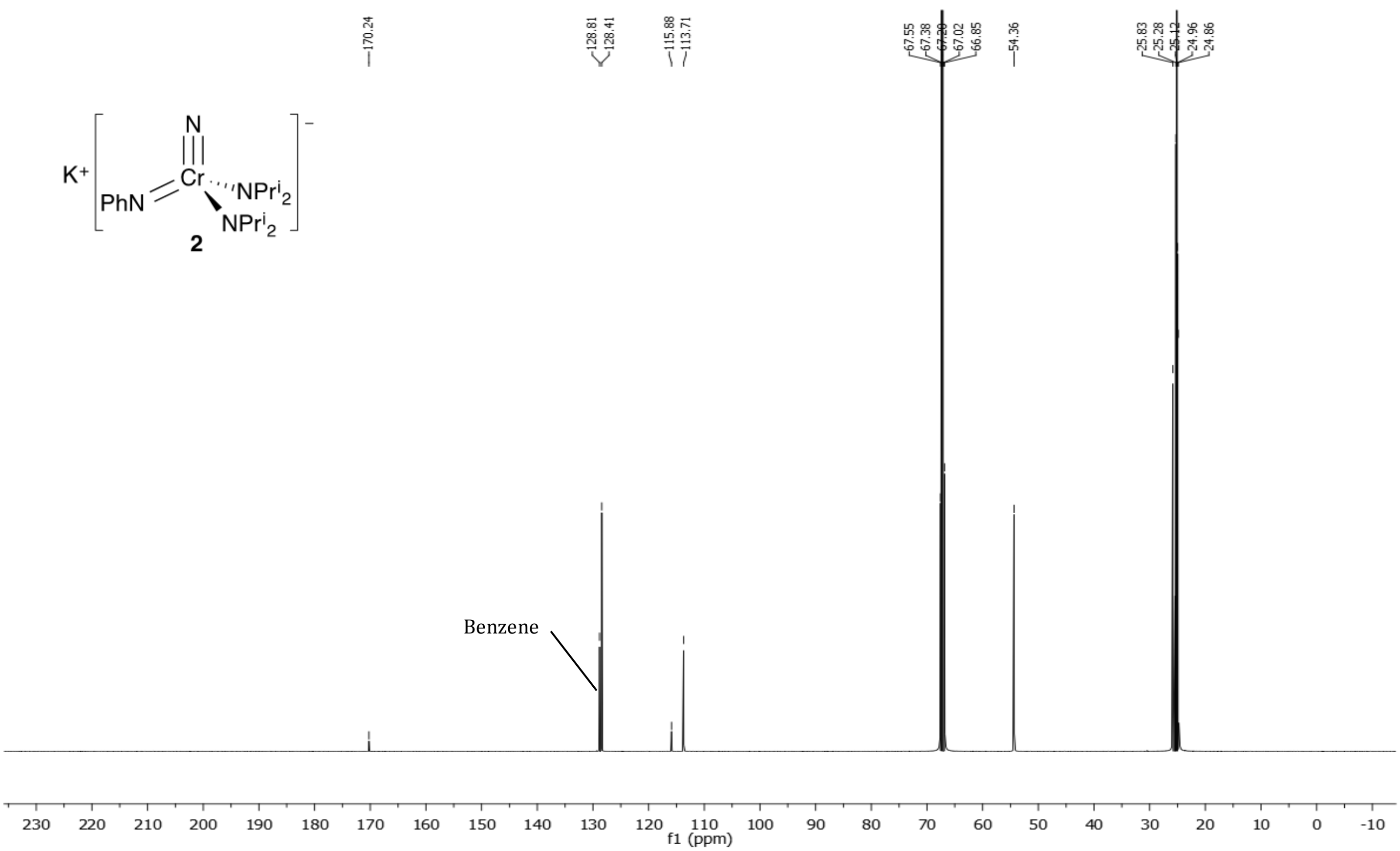

*K{NCr(NPr$^i{}_2$)$_2$(NPh)} (**2**) ^{14}N NMR*

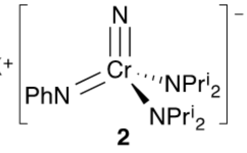

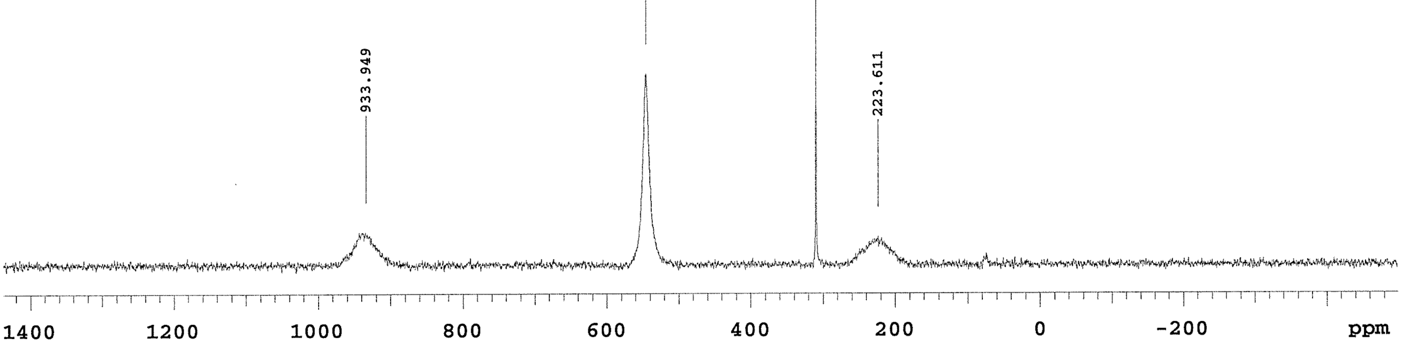

{K(crypt-222)}{NCr(NPr$^i_2$)$_2$(NPh)} ***(CrN123)*** *^{1}H NMR*

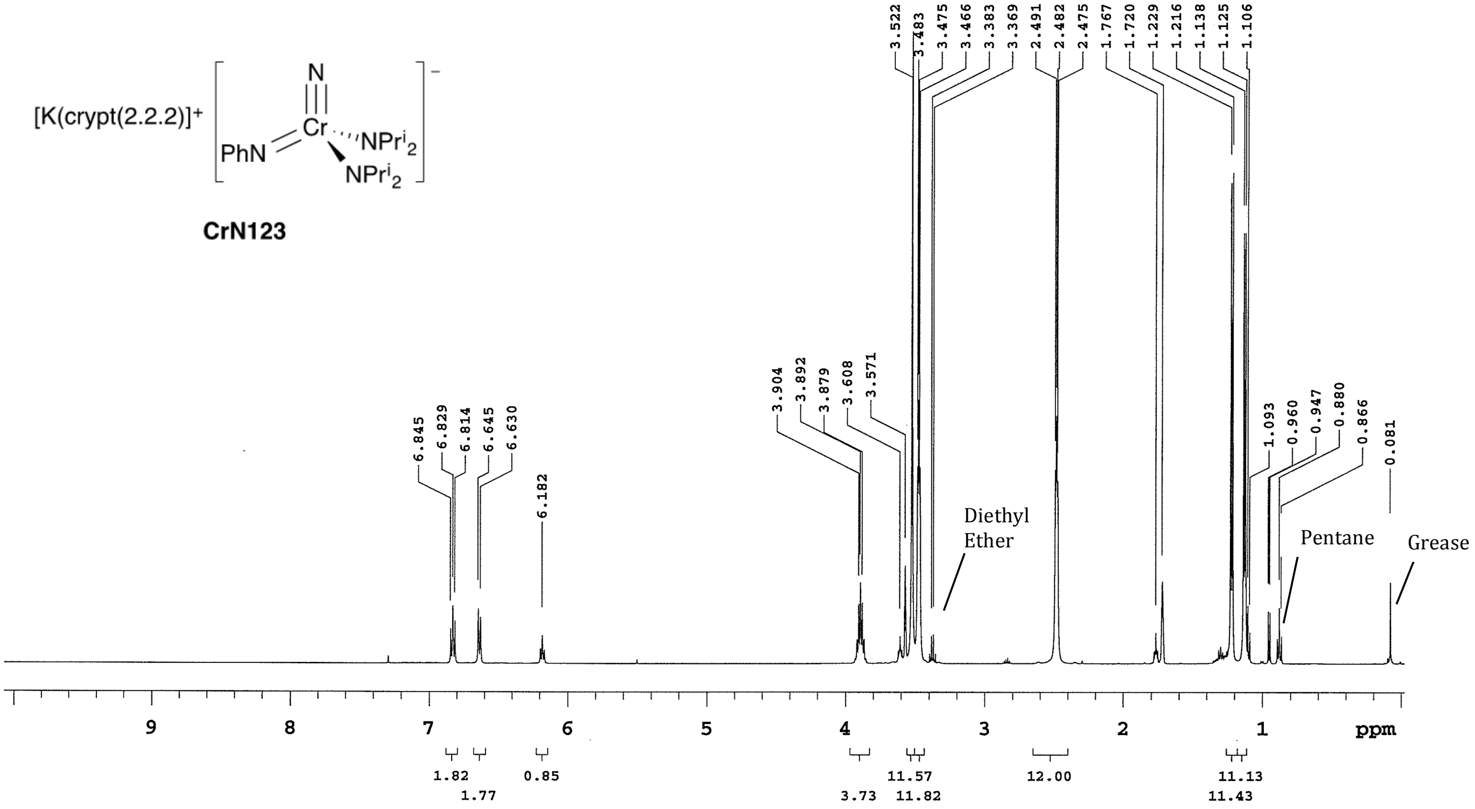

{K(crypt-222)}{NCr(NPr$^i_2$)$_2$(NPh)} ***(CrN123)*** *^{13}C NMR*

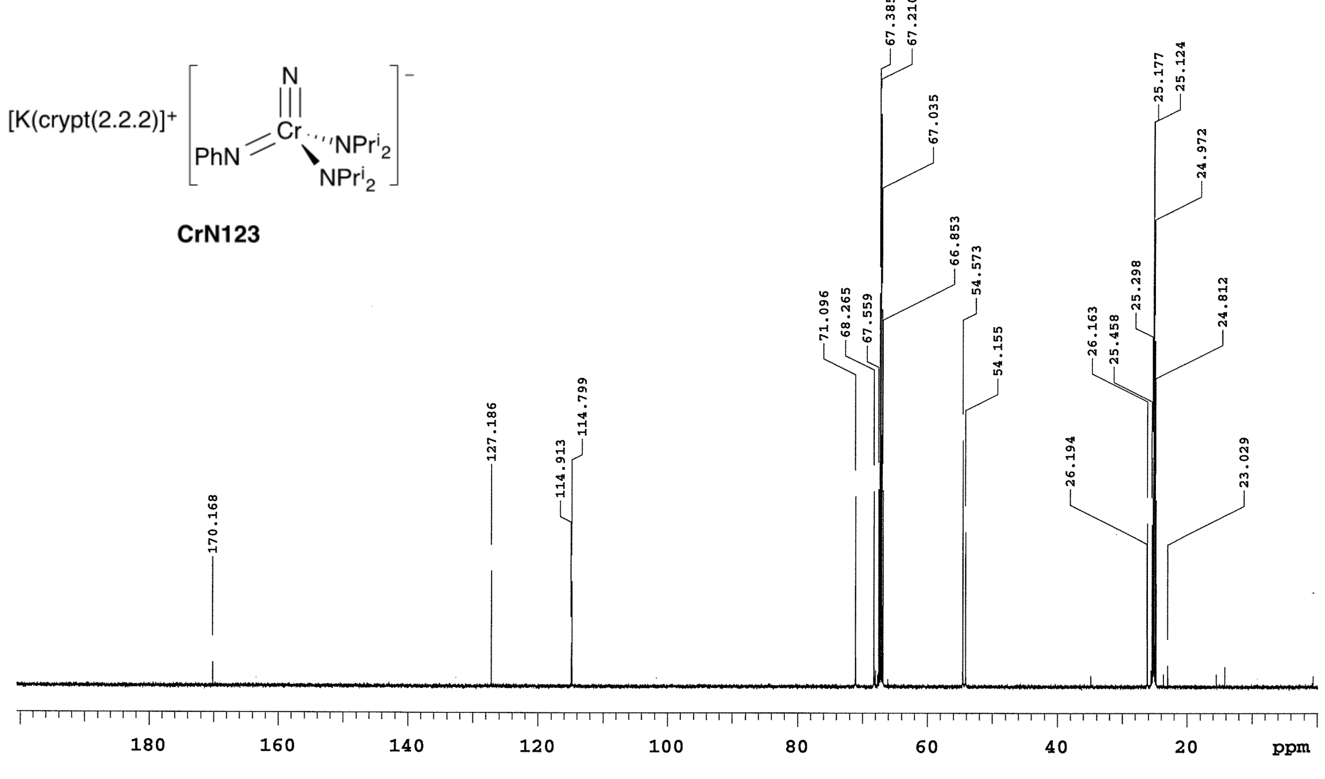

{K(crypt-222)}{NCr(NPr$^i{}_2$)$_2$(NPh)} ***(CrN123)*** *^{14}N NMR*

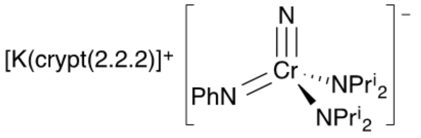

CrN123

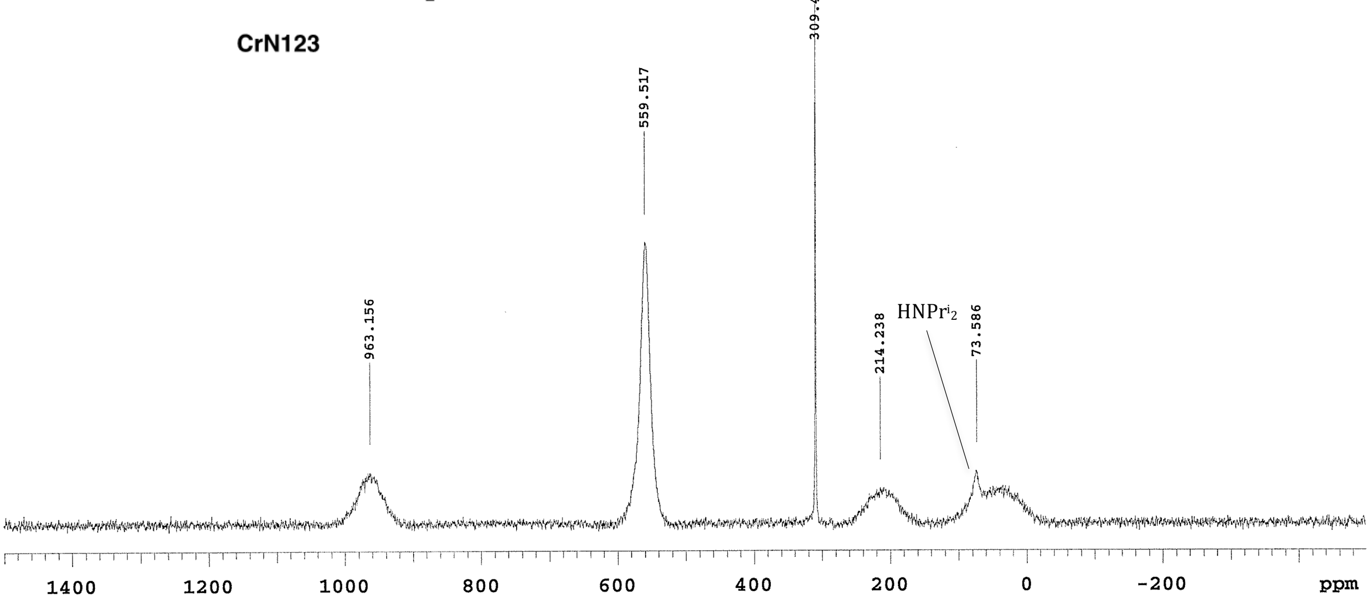

NCr(NPr$^i{}_2$)$_2$(N(Me)Ph) ^{14}N NMR

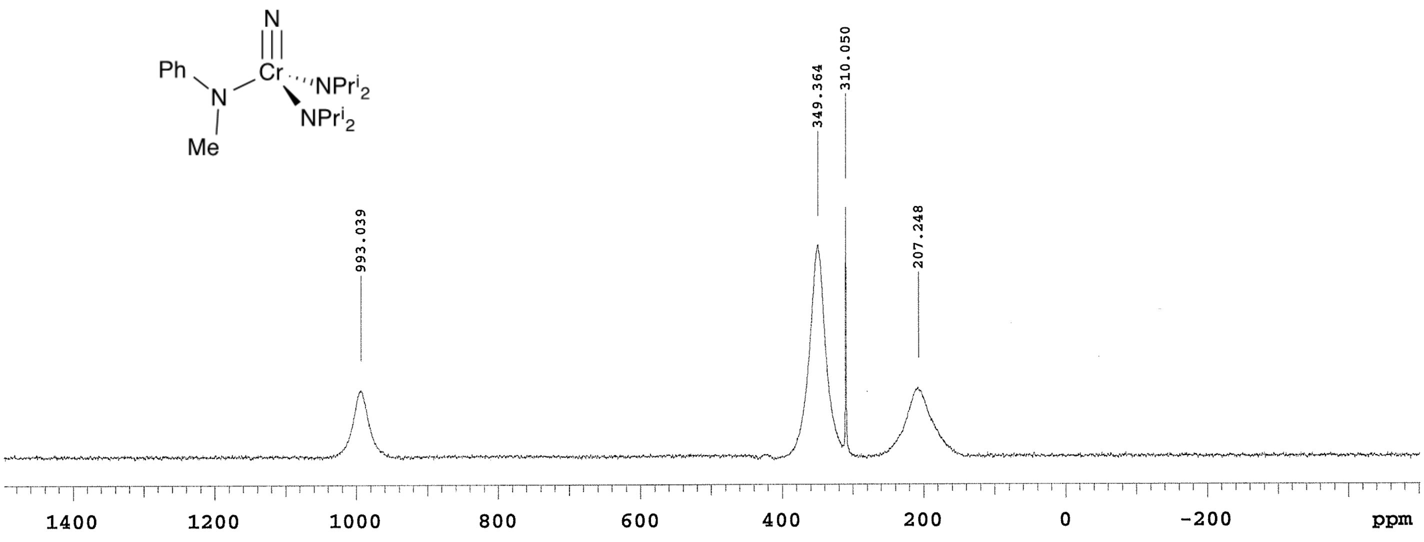

*NCr(NPr$^i{}_2$)$_2$[N(COMe)Ph] (**3**) ^{1}H NMR*

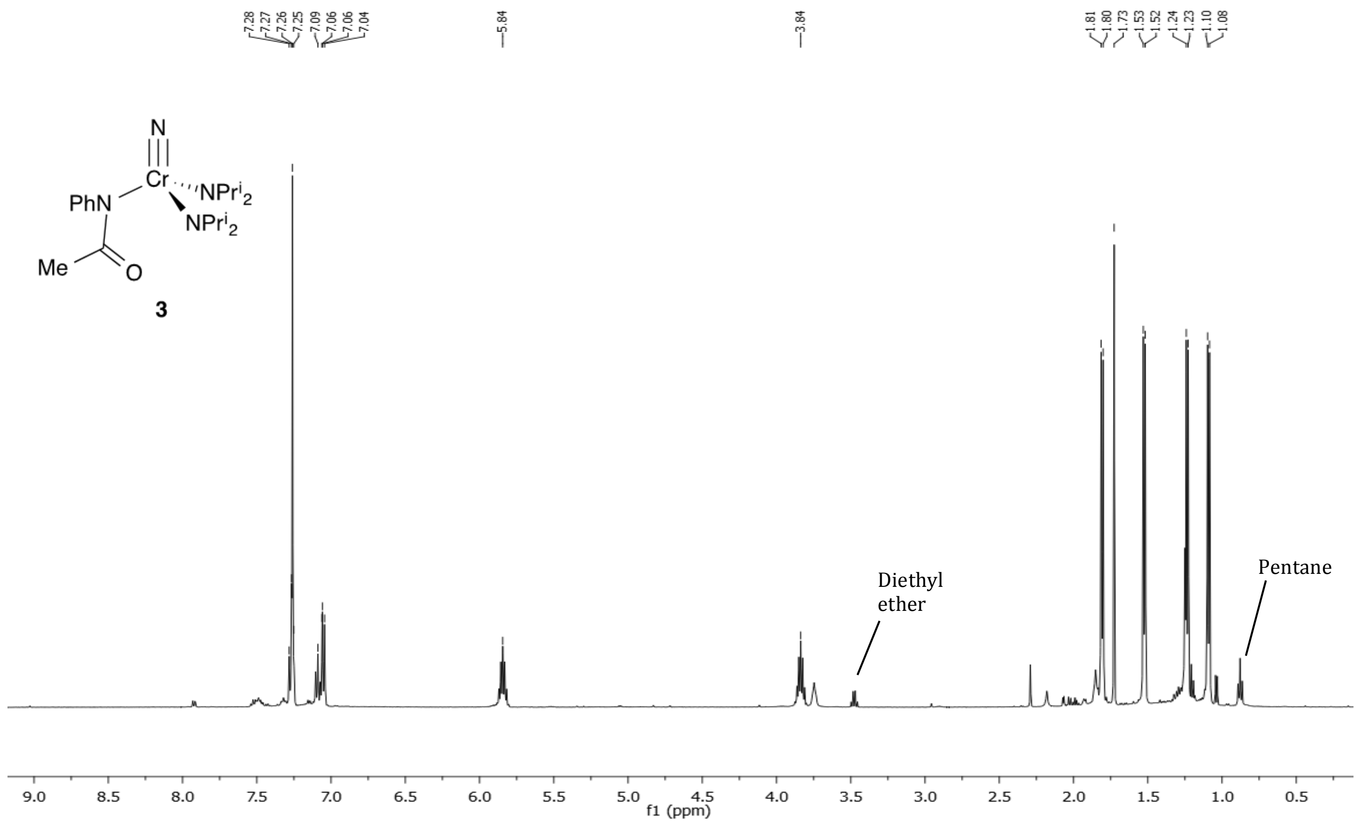

*NCr(NPr$^i{}_2$)$_2$[N(COMe)Ph] (**3**) ^{13}C NMR*

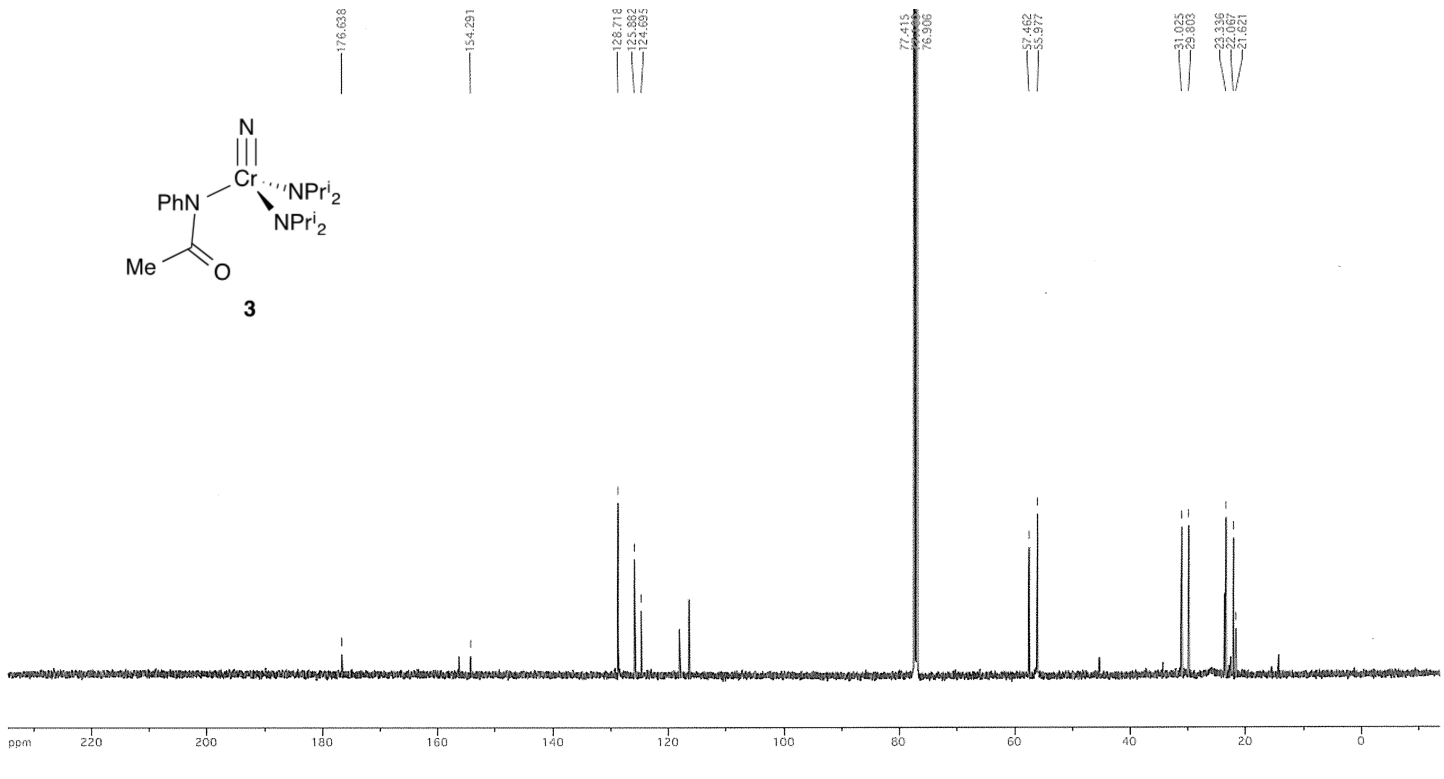

*NCr(NPr$^i_2$)$_2$[N(COMe)Ph] (**3**) ^{14}N NMR*

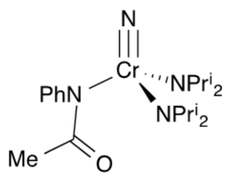

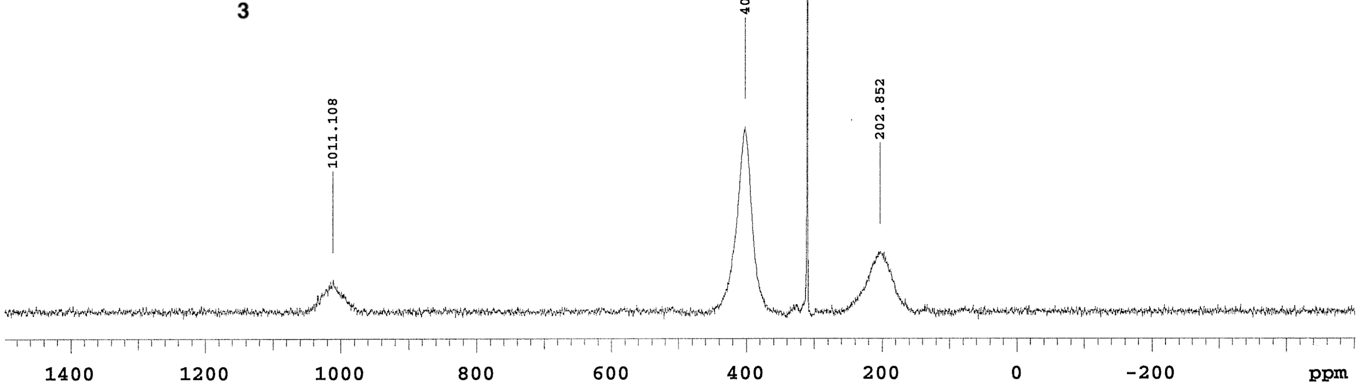

*Cr(NPr^i$_2$)$_2$(NPh)[NC(O)Bu^t] (**4**) ^{1}H NMR*

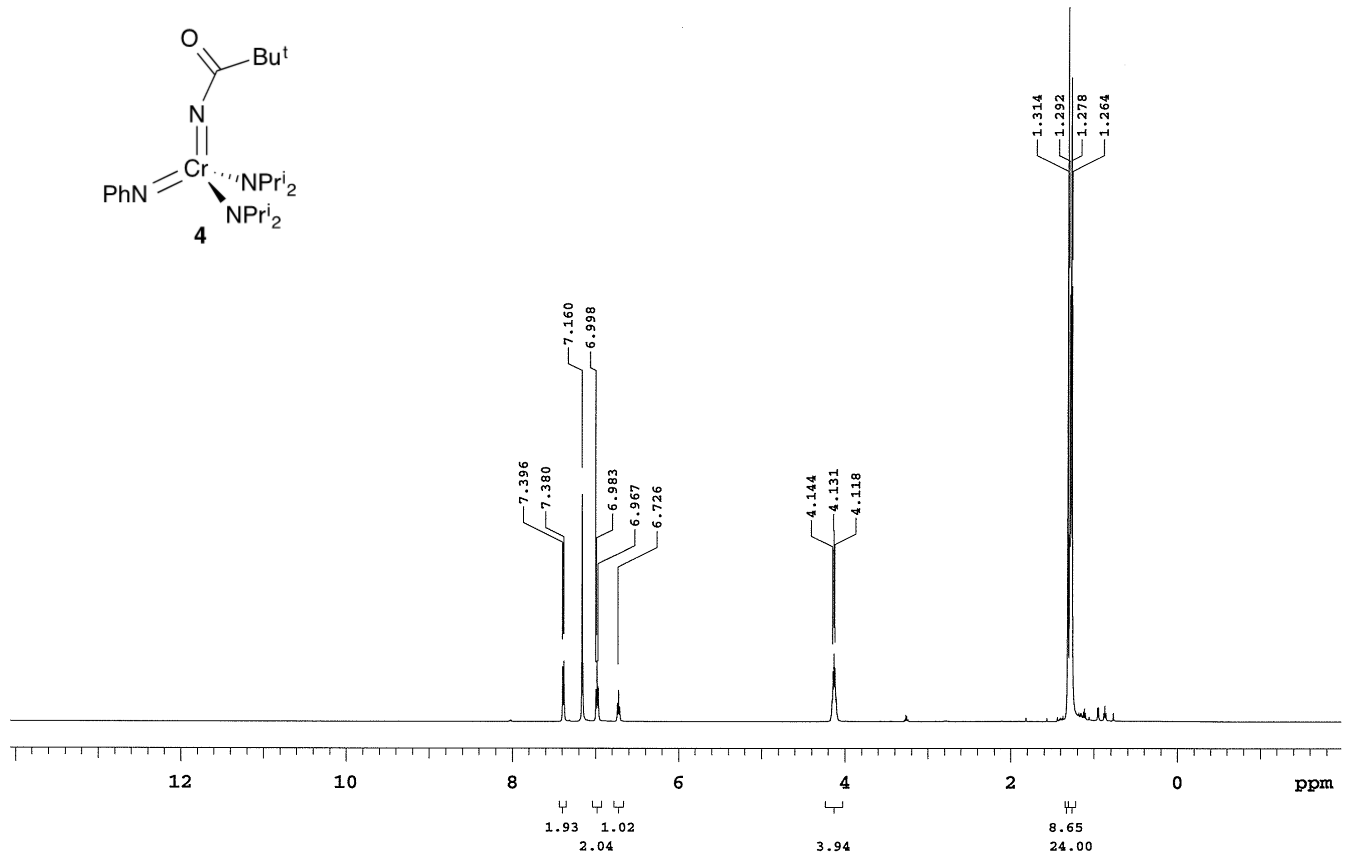

*Cr(NPr^i$_2$)$_2$(NPh)[NC(O)Bu^t] (**4**) ^{13}C NMR*

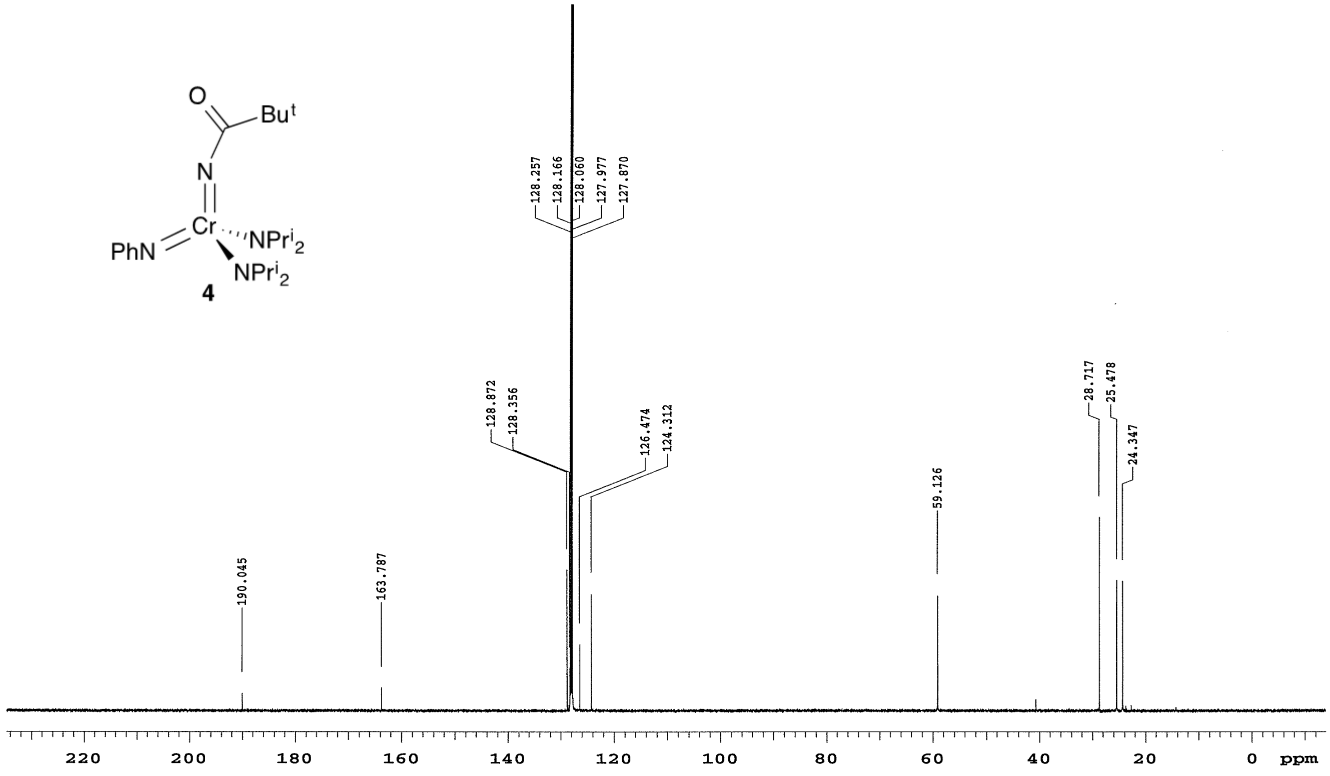

*Cr(NPr$^{i}_{2}$)$_2$(NPh)[NC(O)Bu^{t}] (**4**) ^{14}N NMR*

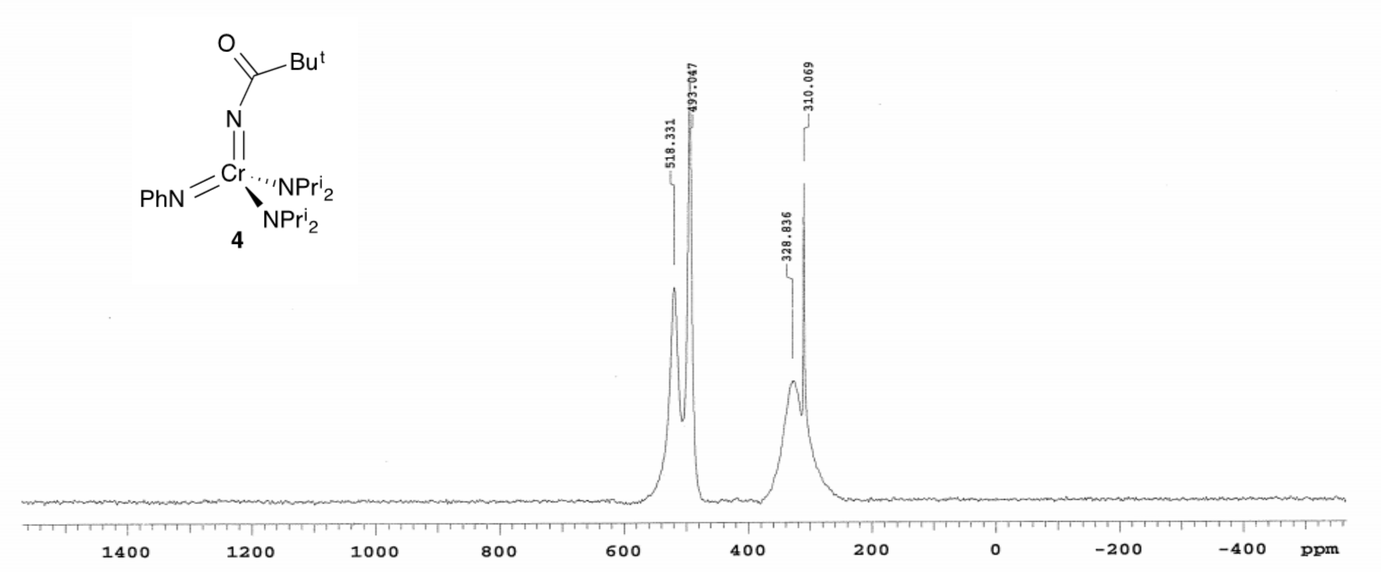

[14]N NMR of Diisopropylamine in THF

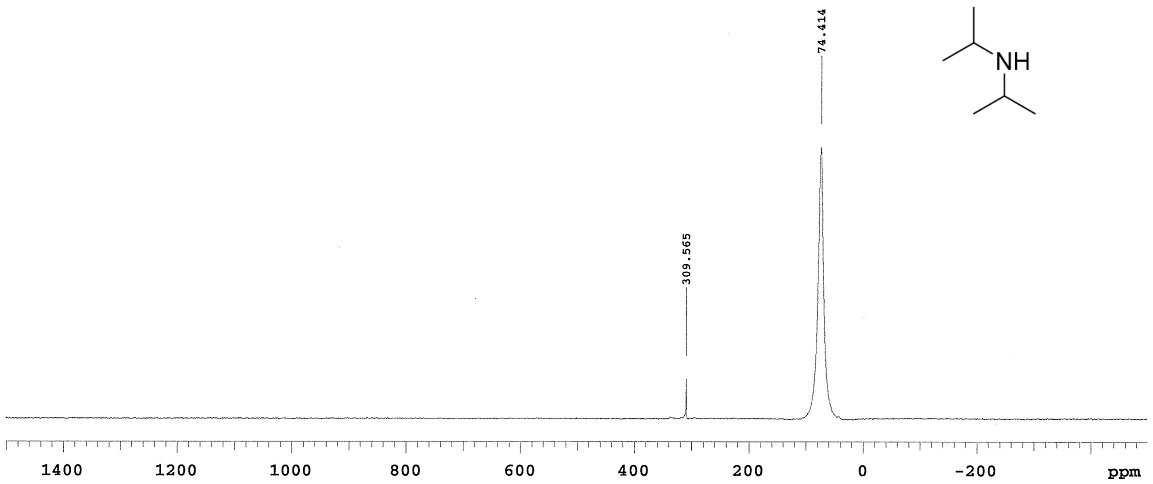

[14]N NMR of 2,2,2-cryptand in $CDCl_3$

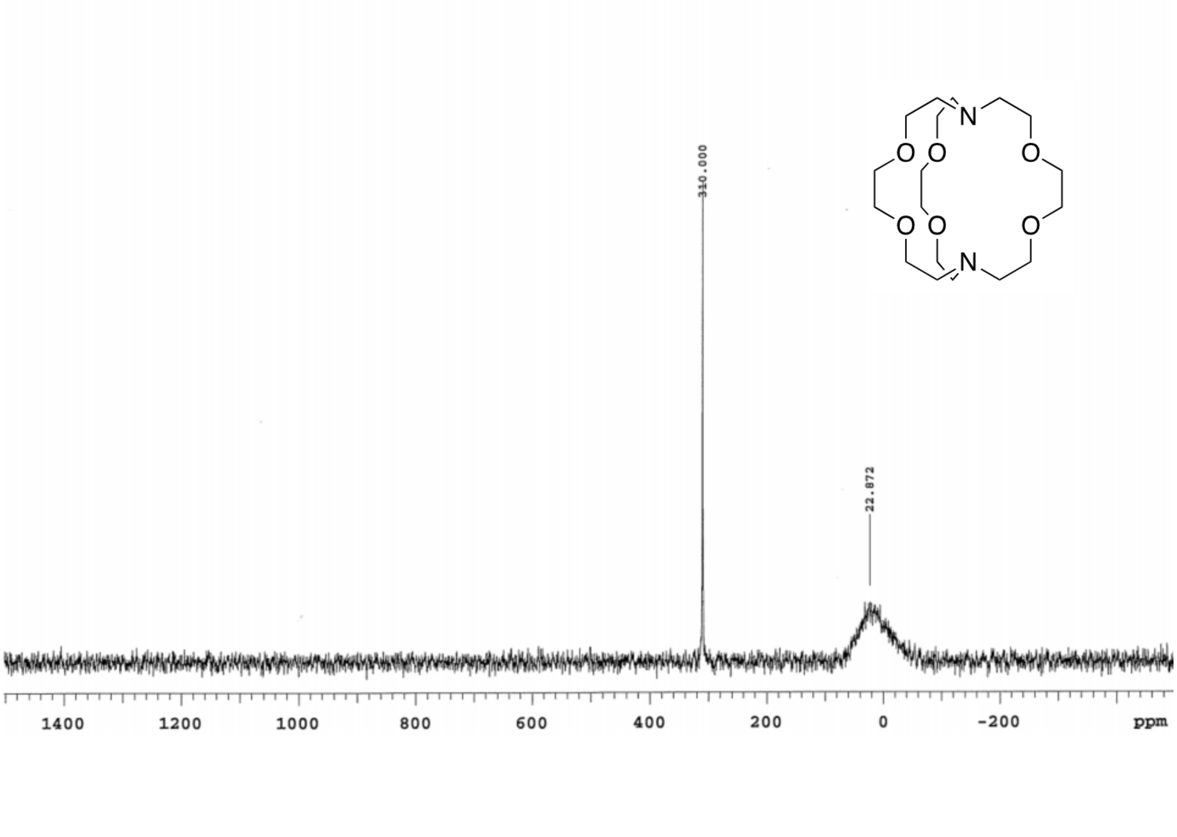

Discussion of Peak Integrations in ^{14}N NMR Spectra

The peak integrals in the ^{14}N NMR spectra listed above and in the manuscript do not necessarily reflect the concentrations on the nuclei in the various environments. The reason for this is that the quadrapolar nucleus of nitrogen relaxes extremely quickly. For example, the nitrogen in the Werner complex $\{Co(NH_3)_6\}^+$ $3Cl^-$ has a relaxation time of a fraction of a millisecond (0.29 ms). Relaxation times of around a millisecond are commonplace for the nucleus, although can be longer in some environments. For example, the terminal nitrogen of N_2O has a relaxation time of 280 ms.[3]

In order to protect the electronics of the NMR probe, there must be a delay between the excitation pulse and the acquisition pulse. For our (aged) instrumentation, the recommended delay for this probe is 0.3 s; consequently, there is some relaxation of nuclei long before we are able to begin our acquisition. Faster relaxing nuclei will appear to have smaller peaks because not all the radiation from their relaxation is being collected, some emission occurs during the delay between pulse and acquisition. Consistent with this, peaks that are sharper correspond to nuclei that generally have slower relaxation times, and the peaks have larger integrals than expected. Peaks that are broader generally corresponded to nuclei relaxing more quickly, and the peaks have smaller integrals than expected.

The fact that the integrals do not correspond to the concentrations of the various nuclei is therefore a product of limitations on our instrumentation.

Method for Determination of Rotational Barriers

The rate constant for the exchange of the two methyne hydrogens of the isopropyl groups was measured using spin saturation transfer (SST) in the ^{1}H NMR. The temperature

chosen for each experiment was based on what was required to reach the slow exchange limit of the complex under investigation, in these cases between −60 °C and 25 °C. T_1 values were measured using the inversion recovery method. Samples were made between 0.02–0.03 M in $CDCl_3$. $\Delta S^{\ddagger}$ for this rotation was shown to be −9 cal•mol^{-1}•K^{-1} for $NCr(NPr^i_2)_2I$ and assumed to be the same for the other compounds. For more details see Reference 2.

Table of LDP Measurements

Table of LDP, SST Measurement Temperature, Rate Constant, and $\Delta G^{\ddagger}$ for $NCr(NPr^i_2)_2X$

X=	LDP (kcal/mol)	Measurement Temperature (°C)	Rate Constant (s^{-1})	$\Delta G^{\ddagger a}$ (kcal/mol)
NHPh	9.56 ± 0.2	–59.0	8.40	11.50
N(Me)Ph	9.57 ± 0.2	–60.0	7.34	11.49
N(COMe)Ph	15.09 ± 0.2	26.7	0.67	17.80

[a] Determined from the rate constant for isopropyl group exchange using the Eyring Equation with the assumption that the transmission coefficient is unity.

Computational Details:

Thermodynamic calculations on the chromium complexes were done using Gaussian09 with B3LYP using the cc-PVDZ basis set and are zero point energy corrected. Mayer Bond Orders were found using the program BORDER v1.0 using calculations from Gaussian09 with the B3LYP functional and SDD basis set on all atoms. NBO calculations are from the DFT results with the cc-PVDZ basis as input to NBO6.

The Natural Resonance Theory bond orders were found on the entire complexes by optimization of the Cr123 complex with cc-PVDZ/B3PW91 and optimization of the W alkylidyne/alkylidene/alkyl complex using SDD/B3PW91. A local NRT calculation was then run with the metal center and only its attached atoms.

References

1. B. S. Billow, R. D. Bemowski, S. A. DiFranco, R. J. Staples, A. L. Odom *Organometallics* **2015**, *34*, 4567-73.
2. S. A. DiFranco, N. A. Maciulis, R. J. Staples, R. J. Batrice, A. L. Odom *Inorg. Chem*. **2012**, *51*, 1187-1200.
3. *Multinuclear NMR*, Joan Mason, Ed., Plenum Press: New York, 1987.
